# Allelism of *Rps3b* and *Rps11* revealed by NLR gene capture of resistance genes to *Phytophthora sojae* in soybean

**DOI:** 10.1002/tpg2.70054

**Published:** 2025-06-12

**Authors:** Yanick Asselin, Luann A. F. Dias, Caroline Labbé, Amandine Lebreton, Vincent‐Thomas Boucher‐St‐Amour, Benjamin Cinget, François Belzile, Gaspar Malone, Francismar C. Marcelino‐Guimarães, Richard R. Bélanger

**Affiliations:** ^1^ Centre de recherche et d'innovation sur les végétaux (CRIV) Université Laval Québec Québec Canada; ^2^ Department of General Biology State University of Londrina Londrina Paraná Brazil; ^3^ Institut de Biologie Intégrative et des Systèmes (IBIS) Université Laval Québec Québec Canada; ^4^ GDM Seeds Cambé Paraná Brazil; ^5^ Brazilian Agricultural Research Corporation National Soybean Research Center (EMBRAPA Soja) Londrina Paraná Brazil

## Abstract

Exploitation of disease resistance genes in soybean (*Glycine max* (L.) Merr.), as an effective method for management of *Phytophthora sojae* (Kauf. & Gerd.), is on the verge of an impasse. Few of the known resistance genes are commercially exploited, and even fewer have been precisely identified. Therefore, little is known about the identities or relationships between those genes, a hindrance preventing optimal introgression of new sources of resistance into elite soybean lines. In this study, we have applied state‐of‐the‐art nucleotide‐binding and leucine‐rich repeat gene capture (RenSeq) using a set of approximately 80,000 unique baits on near‐isogenic lines, whole‐genome resequencing, and bulked segregant analysis to uncover a resistance gene that has remained elusive for 40 years. This work highlights the reassessment of the *Rps3b* locus from Chr13 to Chr7 and the description of two alleles, from Turkish and Chinese landraces, of a sole candidate gene. We have identified *Rps3b* in four, fully resequenced, genetic backgrounds, including the original PI from 1985, in which the resistance gene was originally described. Specificity of the resistant alleles was achieved through phenotypic characterization with field isolates carrying virulent and avirulent forms of the corresponding effector, *Avr3b*. Surprisingly, these alleles showed extremely high synteny and sequence identity with *Rps11* consistent with allelism, and conferred a resistance phenotype indistinguishable from that of the recently cloned *Rps11*. These results offer new sources of resistance for breeders that are effective against the current *P. sojae* pathotypes in the field.

## Core Ideas

- Resistance gene enrichment sequencing (RenSeq) was performed on soybean near‐isogenic lines.
- Localization of *Rps3b* resistance gene in soybean was reassessed from Chr13 to Chr7.
- Two resistance alleles of *Rps3b* were uncovered from Turkish and Chinese landraces.
- *Rps3b* resistance gene is allelic to *Rps11*.
- *Rps3b* conferred a resistance phenotype indistinguishable from that of *Rps11*.

**Abbreviations**
BSA: bulked segregant analysis
CDS: coding DNA sequence
GBS: genotyping by sequencing
NIL: near‐isogenic line
NLR: nucleotide‐binding and leucine‐rich repeat
PRR: Phytophthora root and stem rot
QTL: quantitative trait loci
SNP: single‐nucleotide polymorphism

## 1 INTRODUCTION

Phytophthora root and stem rot (PRR), an important disease affecting soybean (*Glycine max* (L.) Merr.) caused by the oomycete *Phytophthora sojae* (Kauf. & Gerd.), reduces soybean production by almost a million metric tons in the United States and Canada every year (Allen et al., 2017). While the main method of control is the exploitation of resistance genes in commercial cultivars, it is becoming increasingly difficult to choose an effective one. As an example, at least 60% of the isolates present on farms affected by the disease in Manitoba (Canada) could overcome the resistance provided by five different resistance genes (Tremblay et al., 2021). A similar situation is seen in the United States in Michigan, where most *P. sojae* isolates can defeat *Rps1a*, *Rps1c*, and *Rps1k*, three broadly exploited resistance genes (Dorrance et al., 2016). This scenario could rapidly lead to an impasse in terms of disease control because currently, only five *Rps* genes are commercially available to producers in North America.

Known since the 1950s, the genetics of soybean resistance to *P. sojae* has been extensively studied (Bernard et al., 1957). Until now, some 40 genes and alleles conferring resistance to *P. sojae* (*Rps*) have been reported, but very few have been cloned (Clevinger et al., 2024). Among those, two genes from the *Rps1k* locus on chromosome 3 (Chr3) were cloned and shown to induce resistance in transformed lines (Gao & Bhattacharyya, 2008; Gao et al., 2005). More recently, *Rps11* was cloned from a cluster of 12 paralogous nucleotide‐binding and leucine‐rich repeats (NLRs) located on Chr7 and shown to confer resistance to *P. sojae* (Wang et al., 2021). Although numerous genes have been finely mapped, it remains a challenge to establish the true nature or precise localization of the reported *Rps*. This has led to the reporting of a plethora of different resistance genes where uncertainties remain whether they are new, allelic, or simply redundant. Despite this confusion, some chromosomal regions appear to consistently host *Rps* genes such as the ones on chromosomes 3, 7, 13, and 18, regions known to be particularly enriched in NLR genes.

The genetic mapping or allelic test methods have often led to inconsistent and ambiguous results regarding the exact position of such genes as they are usually found in complex loci, thus limiting the discovery of the precise determinants of resistance (Chen et al., 2021). For instance, studies conducted on *Rps3a*, *Rps3b*, and *Rps3c* originally described them as allelic (Athow et al., 1986; Ploper et al., 1985). However, more recent results using molecular markers and recombinant populations failed to confirm these observations. Only *Rps3a* was shown to segregate with markers on Chr13, unlike *Rps3c*, which was rather associated with one marker on Chr18 where *Rps4*, *Rps5*, and *Rps6* reside (Gunadi, 2012). For its part, *Rps3b* was never confirmed through genetic mapping to be located on Chr13. In this context, sequencing‐based methods can offer useful alternatives as they can precisely resolve the complexity of genomic regions hosting numerous resistance genes due to the presence of related paralogous NLR genes. Among those, RenSeq, an NLR gene‐targeted enrichment and sequencing method, has been shown to enable the discovery and annotation of pathogen resistance gene family members in plant genomes (Jupe et al., 2013). It was successfully exploited in species such as *Solanum tuberosum* L. and *Aegilops tauschii* Coss. to identify new resistance genes against *Phytophthora infestans* and *Puccinia graminis* f. sp. *tritici*, respectively (Arora et al., 2019; Witek et al., 2016).

More recently, the method was applied to the soybean–*P. sojae* pathosystem revealing new sources of resistance from Asian accessions (Hodge et al., 2025). Considering the difficulties inherent to the identification of *Rps* genes in soybean, we hypothesized that RenSeq could represent a valuable tool to generate targeted deep sequencing results to uncover previously reported or novel *Rps* genes in soybean.

In this work, an optimized RenSeq approach, using near‐isogenic lines (NILs), was employed to study soybean resistance against *P. sojae*. By using genetic mapping and further confirmation through RenSeq, we show that *Rps3b*, a gene first reported in 1985 (Ploper et al., 1985) and presumed to be allelic to *Rps3a* and *Rps3c*, is in fact located on Chr7 rather than on Chr13. Additionally, whole‐genome long‐read resequencing of four soybean lines known to carry *Rps3b*, including the original PI from 1985, revealed two distinct sources for this resistance including one that shares surprising homology with the newly discovered *Rps11*. Incidentally, hypocotyl inoculation assays showed that *Rps3b‐* and *Rps11*‐resistant lines exhibit identical resistant/susceptible phenotypes matching the allelic form of their corresponding effector, *Avr3b*.

## 2 MATERIALS AND METHODS

### 2.1 Plant materials and *P. sojae* isolates

Seeds of the following *G. max* accessions were obtained from the Germplasm Resources Information Network: Williams (PI 548631), PI 591509 (*Rps3b*), PI 594527 (*Rps11*), and PI 172901 (*Rps3b*). Seeds of Harosoy (PI 548573), as well as those of NILs‐carrying *Rps* genes L88‐1479 (*Rps3b*) and Haro33 (*Rps3b*), were provided by Dr. Owen Wally (Harrow Research and Development Centre, Agriculture and Agri‐Food Canada, Harrow, ON, Canada).

From our collection of *Phytophthora* isolates, 254 isolates retrieved from field sampling across Canada and isolated from soil samples as described by Tremblay et al. (2021) were screened for their *Avr3b* genotype (Table S1) using *Avr3b* allele‐specific primers designed according to the two reference alleles of the gene (Dong et al., 2011). All isolates were fully phenotyped against Williams and Harosoy differentials of resistant NILs. Two representative isolates for each allele of the effector were selected for phenotyping the soybean accessions. Isolates *ULPS_824* and *ULPS_858* were confirmed to carry the avirulent allele (*Avr3b*), and isolates *ULPS_786* and *ULPS_797* were confirmed to carry the virulent allele (*avr3b*).

### 2.2 DNA extraction

All experiments were carried out on gDNA extracted from leaves taken from 10‐day‐old soybean seedlings grown in sterile vermiculite. Leaf samples were homogenized (4 m/s × 2 cycles of 30 s with a 40‐s pause) with an Omni Bead Ruptor 24 (OMNI International), and DNA was extracted using a modified cetrimonium bromide method (Werth et al., 2016). Precautions were taken to maintain the integrity of high molecular weight DNA molecules. DNA concentration was measured on a Qubit 4 fluorometer (ThermoFisher Scientific).

**Core Ideas**

- Resistance gene enrichment sequencing (RenSeq) was performed on soybean near‐isogenic lines.
- Localization of *Rps3b* resistance gene in soybean was reassessed from Chr13 to Chr7.
- Two resistance alleles of *Rps3b* were uncovered from Turkish and Chinese landraces.
- *Rps3b* resistance gene is allelic to *Rps11*.
- *Rps3b* conferred a resistance phenotype indistinguishable from that of *Rps11*.

### 2.3 Bulked segregant analysis (BSA) and quantitative trait loci (QTL) mapping of *Rps3b*

Development of the mapping population and QTL mapping were done at Embrapa Soybean, Londrina, PR, Brazil. The population was obtained by crossing the susceptible Brazilian cultivar BRS 268 with the resistant material PI 591509 (*Rps3b*). All 167 F_2:3_ families and parental lines were grown in a randomized block design composed of three blocks containing 10 plants each (30 replicates per family). This population was assessed for PRR reaction. Note that 11‐day‐old seedlings were inoculated with the *P. sojae* isolate Ps2.4/07 (pathotypes *1d*, *2*, *3c*, *4*, *5*, *6*, and *7*) by the hypocotyl wound‐inoculation technique with some modifications (Costamilan et al., 2013; Schmitthenner et al., 1994). Seedlings were classified as follows: (1) resistant when they presented no disease symptom; (2) moderately resistant when they presented a brown infection lesion around the inoculation slit; and (3) susceptible when they presented a brown lesion at the point of infection and the plant died after a few days. DNA extraction and BSA were done on grouped individuals based on their F_2:3_ phenotypes (Keim, 1988; Michelmore et al., 1991). Two resistant and two susceptible DNA bulks were made by mixing equimolar amounts of DNA from seven different plants each. The DNA of each bulk and one replicate of each parental variety were genotyped by sequencing (Ion Torrent) approximately 2000 single‐nucleotide polymorphisms (SNPs) distributed throughout the soybean genome using a custom AgriSeq tGBS (where GBS is genotyping by sequencing) panel (Thermo Fisher Scientific) at the GDM Molecular Biology Laboratory, Londrina, PR, Brazil. The genotypic data were analyzed for selection of SNPs co‐segregating accordingly between parental varieties and bulks. The intervals defined by the SNPs that consisted of positive hits in the BSA were selected for saturation as well as the interval where *Rps3a* and *Rps3b* were previously mapped (Gunadi, 2012). Genomes of both parental varieties were compared using Illumina NGS technology (approximately 10x depth for BRS 268 and 30x depth for PI 591509), and polymorphic SNPs between the parents in the target intervals were kept (Table S2). These markers were then used to genotype the parents and the F_2_ population by the PlexSeq tGBS approach at Agriplex Genomics or by KASP (LGC Group). The markers were named after their genomic position in the reference genome Williams82‐v2 (Song et al., 2016). Phenotypes of F_2:3_ families were then scored as “resistant” and “moderately resistant,” resulting in two traits for mapping: “percentage of resistant plants—PR” and “percentage of resistant plus moderately resistant plants—PRM.” These traits were mapped by composite interval mapping (CIM), performed in the software QTL IciMapping version 4.2 (Meng et al., 2015). The significance threshold for CIM (*α* = 0.05) was determined by performing 1000 permutations. The percentage of the variance explained, as well as the chi‐square (*χ*2) tests for the segregation distortion for each molecular marker following the hypothesis of a 1:2:1 segregation ratio, was also calculated by the software.

### 2.4 Bait design

NLR baits were designed using the web version of the Multiple Em for Motif Elicitation (MEME) suite (Bailey et al., 2015). First, the MEME tool was used in discriminative mode to search for 20 amino acid motifs characteristic of NLRs on Jupe et al. (2012) training sets. The “positive” set was composed of 53 characterized NLR genes from diverse plant species plus six additional NLRs from *G. max* and *Glycine soja* Siebold & Zucc. The “negative” set contained 16 nucleotide‐binding proteins and pattern recognition receptors. The motifs found with MEME were queried with a Motif Alignment & Search Tool search on the complete protein dataset derived from the Williams82‐v4 reference genome (Valliyodan et al., 2019). Genes were considered to be NLRs if their *e*‐value was lower than the lowest *e*‐value from the “negative” set. The open reading frames of all genes respecting the cutoff value were extracted along with 2 kb of flanking region both up‐ and downstream. A similar approach was conducted on the *G. soja* W05 reference genome, but only the coding DNA sequence (CDS) of candidate NLRs was kept for bait design to increase diversity in the bait set. Nucleotide sequences of all candidate NLR genes were sent to Daicel ArborBioscience for bait production. The complete bait library consisted of 78,022 unique 100‐mer oligos providing twofold coverage of the gene set because of a 50‐nucleotide overlap between the baits.

### 2.5 NLR gene capture

#### 2.5.1 Short‐read capture

The DNA concentration of Williams (PI 548631), Harosoy (PI 548573), L88‐1479 (*Rps3b*), and Haro33 (*Rps3b*) was normalized to 10 ng/µL prior to fragmentation to a mean length of 450 bp using a Covaris M220 (Covaris, LLC.) (*T*° = 20, *t* = 100 s, peak power = 50 W, duty factor = 20, cycle/burst = 200) in microTUBEs AFA fiber screw cap. Fragment length distribution was measured using an Agilent 2100 Bioanalyzer (Agilent Technologies). Illumina libraries were prepared from sheared gDNA using the NEBNext Ultra II DNA Library Prep Kit from New England BioLabs. DNA was end‐repaired, and an A overhang was added. Size selection was conducted using AMPure XP beads (Beckman Coulter) with two consecutive clean‐ups (0.15x and 0.1x ratios) to select for insert sizes between 500 and 700 bp. Libraries were individually indexed with Illumina Truseq HT dual‐indexed adaptors and PCR (polymerase chain reaction) amplified for eight cycles according to the manufacturer's protocol. A final reaction clean‐up was executed on the amplified libraries using AMPure XP beads and a 0.9x ratio. Appropriate (500 bp) library size distribution was confirmed using an Agilent 2100 Bioanalyzer.

For each capture reaction, eight samples were pooled together (250 ng per library) for a total of 2 µg per enrichment reaction. Baits were added to each pooled library, and the whole reaction was incubated at 65°C for 20 h to allow hybridization with target molecules. Baits (and their targets) were then retained by streptavidin‐bound metallic beads, and all unhybridized DNA molecules were washed away. Captured molecules were PCR amplified with KAPA HiFi HotStart Ready Mix (Kapa Biosystems, Inc.) and the Illumina P5 and P7 library primers for 14 cycles. The final reaction was cleaned using a 0.7x bead ratio and sent for paired‐end sequencing on an Illumina MiSeq system for a read length of 300 bp.

#### 2.5.2 Long‐read capture

Precautions were taken to maintain the integrity of high molecular weight DNA molecules throughout the procedure. DNA concentration was normalized to 20 ng/µL and a volume of 200 µL prior to fragmentation to a length of 3000 bp using a Covaris M220 (*T*° = 20, *t* = 600s, peak power = 8 W, duty factor = 20, cycle/burst = 1000) in redMinitubes by Covaris. Purification and size selection were done using AMPure XP beads with a ratio of 0.4x according to the manufacturer's protocol.

Fragment length distribution was evaluated using the QIAxcel Advanced System from Qiagen. Illumina libraries were prepared on fragmented DNA using the NEBNext Ultra II DNA Library Prep Kit from New England BioLabs. Each soybean line was barcoded using NEBNext Multiplex Oligos for Illumina sequencing. Libraries were then purified using AMPure XP beads with a ratio of 0.5x according to the manufacturer's protocol. All libraries were PCR amplified using the KAPA HiFi HotStart Ready Mix and the Illumina P5 and P7 library primers with a 4‐min extension time for eight cycles. A final purification step was done on amplified libraries using AMPure XP beads with a ratio of 0.4x according to the manufacturer's protocol.

Illumina libraries were pooled in groups of three, consisting of 83 ng of DNA per sample for a total of 250 ng, and were mixed with the baits for 20 h at 65°C to allow hybridization with DNA molecules. Baits and their targets were then retained by streptavidin‐bound metallic beads, and all unhybridized DNA molecules were washed away. All captured molecules were PCR amplified using the KAPA HiFi DNA Polymerase and the Illumina P5 and P7 library primers with a 4‐min extension time and 25 cycles. A final step of purification was done on amplified libraries using AMPure XP beads with a ratio of 0.4x according to the manufacturer's protocol.

A second library preparation step was carried out on captured molecules for Nanopore sequencing. The ligation sequencing kit (SQK‐LSK109) and Native Barcoding expansion 1–12 (EXP‐NBD104) were used according to the manufacturer's protocol. Finally, samples were all pooled in one bulk and loaded on two flow cells (FLO‐MIN106) to be sequenced on a MinIon device from Oxford Nanopore Technologies.

### 2.6 Bioinformatic protocol for candidate gene identification

#### 2.6.1 Data processing

Quality control and read trimming were executed on CLC Genomics Workbench from Qiagen for both capture experiments. Only Illumina reads meeting a Phred score of 20, devoid of ambiguities, and longer than 150 bp were kept. Raw Nanopore Fast5 sequences were basecalled into fastq using Guppy 5.0.7, and barcodes were trimmed. Because three samples were barcoded with the same Nanopore index, a second round of demultiplexing was done. Guppy parameter files were modified to include the sequence of Illumina barcodes used in the experiment. Illumina barcodes were trimmed by removing 70 bp from the 5′ and 3′ ends of each read. Thereafter, Porechop 0.2.4 was used to confirm no adaptors were left in the dataset. Reads were filtered to retain those longer than 500 bp and with a Phred score higher than 7.

#### 2.6.2 Assemblies

At first, NLRs from recurrent parents (Harosoy and Williams) were assembled using Canu 2.1.1 (genome size = 40 M, rawErrorRate = 0.5, correctedErrorRate = 0.144, minReadLength = 1000, minOverlapLength = 500) (Koren et al., 2017). Then, draft assemblies were polished three times with their respective long‐read dataset using Racon 1.3.1. Then, Medaka 1.4.3 was used to correct draft assemblies with default parameters. Finally, parental assemblies were polished four times using Pilon 1.23 with Illumina reads (Walker et al., 2014).

#### 2.6.3 Candidate gene identification

To identify candidate genes for *Rps3b*, NLR‐based comparisons between resistant NILs and their recurrent parents were performed. Illumina reads from Haro33 (*Rps3b*) and L88‐1479 (*Rps3b*) were mapped on the NLR assemblies obtained for their respective parents, Harosoy and Williams.

To control misassembled NLR genes, Illumina reads from Harosoy and Williams were mapped on their own NLR assemblies, and SNP variants were called. Because a perfect match should occur between a dataset and its corresponding genome, only misassembled contigs, sequencing errors, or heterozygosity could explain those variants. Then, variants found for the recurrent parents were filtered out from the SNPs identified between the NILs and their recurrent parent so that only unique SNPs were kept for the identification of candidate genes. Finally, all residual variants were plotted against the chromosomal positions on the Williams82‐v4 soybean reference genome (Valliyodan et al., 2019) to generate a dot plot.

#### 2.6.4 Whole‐genome sequencing and synteny analysis

Soybean accessions PI 591509 (*Rps3b*), PI 172901 (*Rps3b*), L88‐1479 (*Rps3b*), and Haro33 (*Rps3b*) were sequenced on a MinIon device from Oxford Nanopore Technologies. Libraries of each line were sequenced on two separate flow cells (FLO‐MIN106) to obtain good coverage over the genome. Genomes were assembled using Canu 2.1.1 (genome size: 1 Gb, rawErrorRate = 0.5, correctedErrorRate = 0.144, minReadLength = 1000 bp, minOverlapLength = 500 bp) and then were polished four times using Pilon 1.23 with RenSeq Illumina reads. Contigs containing the candidate region of Chr7 were retrieved from the assemblies and compared using the Python‐based MCscan (v1.1.8) for synteny analysis.

### 2.7 Phenotyping and *Avr3b* recognition

Response to *P. sojae* infection was assessed using the hypocotyl wound‐inoculation method (Dorrance, 2018). Resistant soybean lines L88‐1479 (*Rps3b*), Haro33 (*Rps3b*), and PI 594527 (*Rps11*) alongside susceptible (Williams and Harosoy) controls were confronted with avirulent (*ULPS_824* and *ULPS_858*) and virulent (pathotype *3b*) (*ULPS_786* and *ULPS_797*) *P. sojae* isolates. Briefly, tested lines were sown in 8‐cm pots. After 7 days, a 1‐cm incision was performed 1 cm below the cotyledonary node using a scalpel while making sure the blade did not go past the xylem. The inoculum consisted of 7‐day‐old *P. sojae* culture grown in V8‐juice agar. The media was cut and transferred into a 10‐mL syringe to be applied inside the longitudinal cut. Each pot consisted of 11 plants per pot, with 10 for inoculation and one as a control. The control plant was marked with tape, wounded, and inoculated with clean V8‐juice medium. All pots were placed in a tray covered by a 6.5‐inch dome and watered from the bottom. The trays were placed in a growth chamber at 24°C, and a black plastic bag was placed over the dome for 48 h. At 5 dpi, disease was rated according to the number of dead plants. Soybean lines were considered resistant if the plants in a pot showed <30% of mortality and susceptible if >70% died from the infection as per Dorrance et al. (2008). We conducted the phenotyping experiments on three separate occasions.

## 3 RESULTS

### 3.1 *Rps3b* segregates with markers on chromosome 7

**FIGURE 1:**
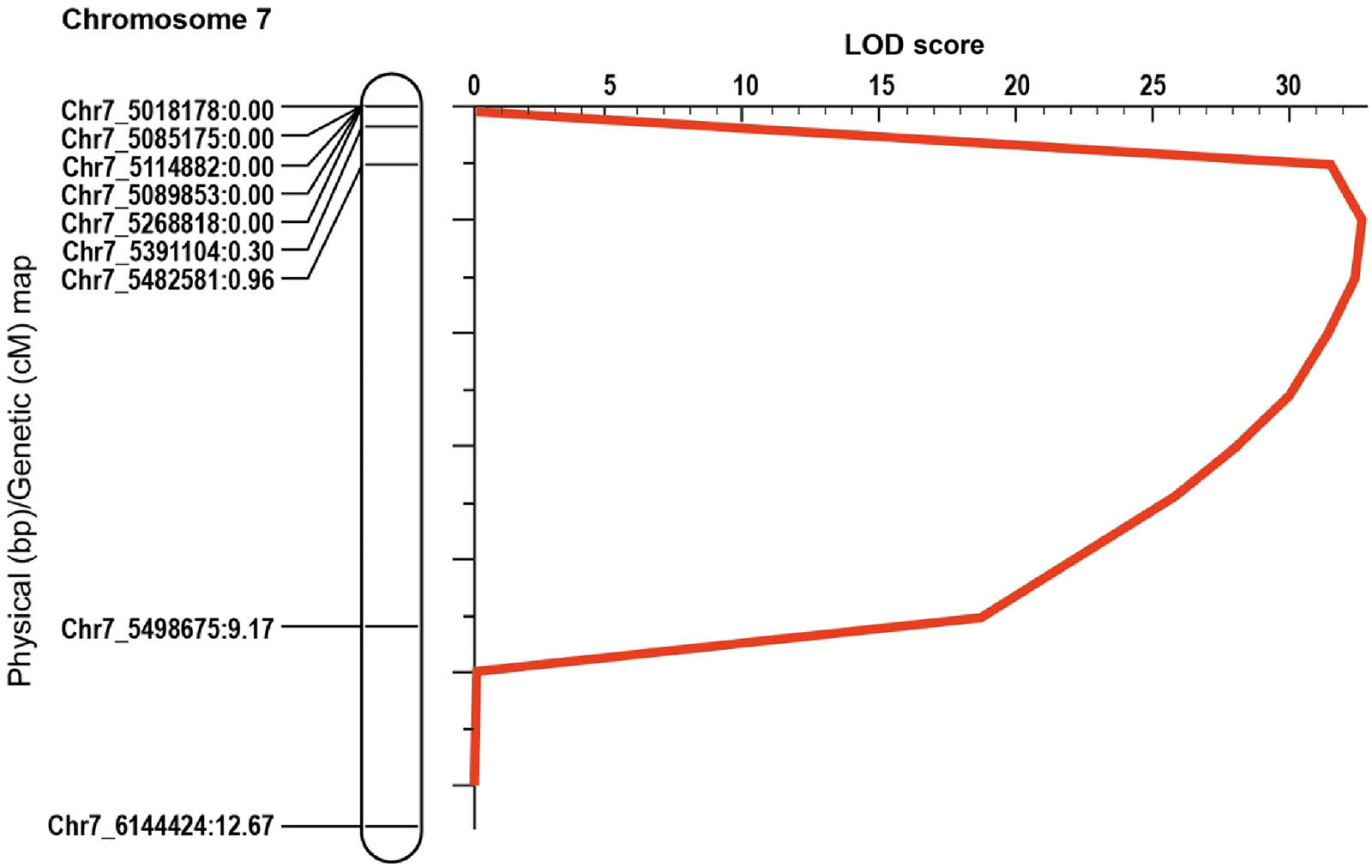
Mapping of quantitative trait loci (QTL) for resistance to *Phytophthora sojae* derived from soybean PI 591509 (*Rps3b*) on Chr7. Mapping from a population of 167 F_2_ derived from a cross between BRS 268 and PI 591509. Marker identification and genetic position (cM) are shown on the left. The curve indicates the physical position of markers against the LOD score of the QTL detected.

To determine the position of the *Rps3b* gene, F_2:3_ homozygous families from a cross between PI 591509 (*Rps3b*) and BRS 268 (susceptible) were assembled into two resistant and two susceptible bulks based on their respective phenotypes. Genotyping of the four bulks and the two parental varieties identified 1192 markers that were polymorphic between the two parental lines (among ∼2000 tested). BSA found positive associations between genotypes and phenotypes among bulks and parental varieties for six SNPs (Table S2). One of them was located on Chr3, at position 2,971,986 bp, close to a region where several *Rps* genes are found. Three of them were located on Chr7 at positions 6,463,180 bp, 8,028,697 bp, and 9,233,014 bp (2.77‐Mb interval), 1–4 Mb away from the *Rps11* locus. The last two were located closely on Chr17 at positions 7,744,589 bp and 7,780,377 bp, more than 23 Mb apart from the closest *Rps* gene (Zhang et al., 2013). Since those SNPs were heterozygous in the F_2_ population (F_3_ phenotypes) and their extreme distance from other *Rps* genes, Chr17 was not included in subsequent mapping analysis. To refine the analysis, genotyping of the entire mapping population was performed with a more extensive set of markers (Table S3) located in the candidate regions of Chr3 and Chr7 identified via BSA. Markers from the *Rps3a* region on Chr13 were also included. QTL analysis failed to detect any association with markers on Chr3 and Chr13 but detected a strong signal in a single 500‐kb interval (5–5.5 Mb; ∼10 cM) of Chr7. This interval produced an LOD (logarithm of the odds) score of 32.7 and explained 59.4% of the variance of the resistant bulk (Figure 1). Based on these results, it was concluded that the *Rps3b* locus only segregated with markers on Chr7 since no marker from Chr13 showed any association with the resistance provided by *Rps3b*.

### 3.2 RenSeq narrowed the *Rps3b* locus to a 200‐kb region of chromosome 7

For the capture and sequencing of NLR genes from the resistant NILs and their recurrent parents, RenSeq baits were developed from 524 *G. max* full‐length genes (including introns) and CDS of 243 *G. soja* genes. The baits were designed to capture the whole NLR gene diversity within *G. max* and *G. soja*, a target covering about 4.8 Mb or 0.48% of their genome. Between one and two million Illumina 300‐bp reads were generated for Haro33 (*Rps3b*), L88‐1479 (*Rps3b*), Harosoy, and Williams, representing a coverage of 60x–120x over the reference gene set. After enrichment and sequencing, 75% of the reads were mapped to NLR genes, which represents a 150‐fold enrichment in NLR‐associated reads. Long‐read RenSeq using Oxford Nanopore Technologies performed on the same resistant lines and recurrent parents generated an average of 800 Mb per sample, corresponding to a coverage of 160x of the reference gene set.

**FIGURE 2:**
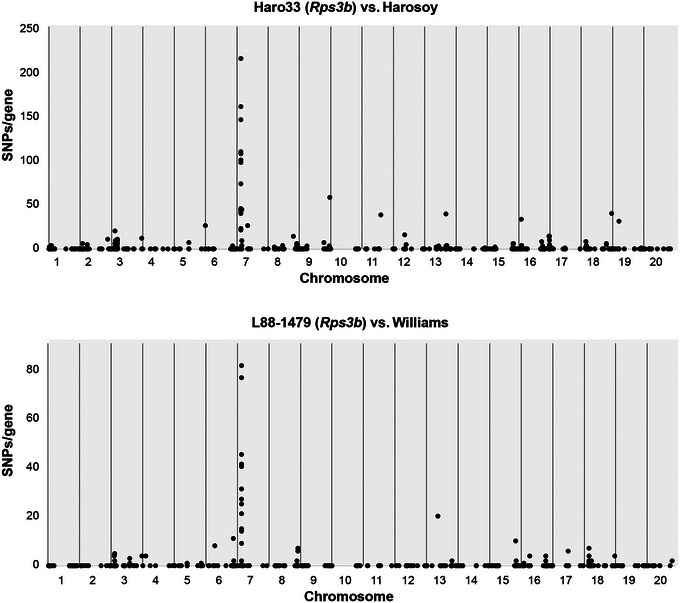
*Rps3b* candidate region mapping through RenSeq. Dot plot graph of nucleotide‐binding and leucine‐rich repeat (NLR) comparisons between Haro33 (*Rps3b*) and L88‐1479 (*Rps3b*) soybean resistant lines to *Phytophthora sojae* pathotype *3b* against their respective recurrent parents Harosoy and Williams. Each parental NLR is represented by a dot and placed according to its position on the soybean reference genome, and the number of single‐nucleotide polymorphisms (SNPs) between the resistant NIL and its recurrent parent is shown along the *y* axis.

As NILs share most of their genome with their respective recurrent parent, we exploited this characteristic to impute which NLR gene belonged to which parent so that only the ones inherited from the resistant parent would become candidates for *Rps3b*. Using long and short RenSeq reads of recurrent parents Harosoy and Williams, 816 and 739 contigs containing NLR‐associated motifs were respectively assembled and employed as a reference for mapping Illumina RenSeq reads of resistant Haro33 (*Rps3b* in Harosoy background) and L88‐1479 (*Rps3b* in Williams background). Not all NLR genes were assembled into one contig because the baits were not designed to capture the large introns they sometimes contain. Variants were called and filtered to remove the ones related to misassemblies. A dot plot illustrating the genomic position of parental NLR genes against variants found in the resistant NIL was generated for each pair of lines. If two genes matched, nearly no variants should be observed, whereas if they diverged, several SNPs would be seen, thus revealing all NLR genes inherited from the resistant parent. In both comparisons, a strong, unique, and similar candidate region was highlighted between 5 and 6 megabases on Chr7 (Figure 2). Only this group of NLRs showed an absence of homology between resistant NILs and their recurrent parent. This cluster corresponds to the eight NLR genes present between positions 5,536,000 and 5,750,000 bp on Chr7 of the Williams82‐v4 reference genome. Even though RenSeq showed that the resistant NILs carried distinctive NLR genes, absent from their recurrent parent, whole‐genome sequencing was carried out to confirm their exact sequences and organization.

### 3.3 Whole‐genome resequencing reveals two alleles of *Rps3b*

To anchor the *Rps3b* candidate region into the soybean genome, whole‐genome resequencing of resistant lines L88‐1479 (*Rps3b*), Haro33 (*Rps3b*), PI 591509 (*Rps3b*), and PI 172901 (*Rps3b*) using Oxford Nanopore Technologies was performed. The sequencing runs allowed the complete and gap‐free assembly of the full candidate region including 300 kb up‐ and downstream for a total of 1 Mb. From these four lines, two syntenic NLR clusters were revealed at the resistance locus on Chr7, one from L88‐1479, comprising 12 NLR genes spanning over 470 kb, and the other from Haro33, PI 591509, and PI 172901, harboring 17 NLR genes covering 700 kb. In the 17‐gene locus of these three lines, all predicted NLR proteins possessed a coiled‐coil domain (CC‐NLR) in their N‐terminus and ranged from 1871 to 2933 amino acids. Importantly, not a single SNP was observed among these lines within the coding regions of these 17 genes. The cluster present in L88‐1479 also contained CC‐NLRs ranging from 1847 to 2464 amino acids. Unexpectedly, this whole region was also found at the same position in the publicly available PI 594527 (*Rps11*) genome.

**FIGURE 3:**
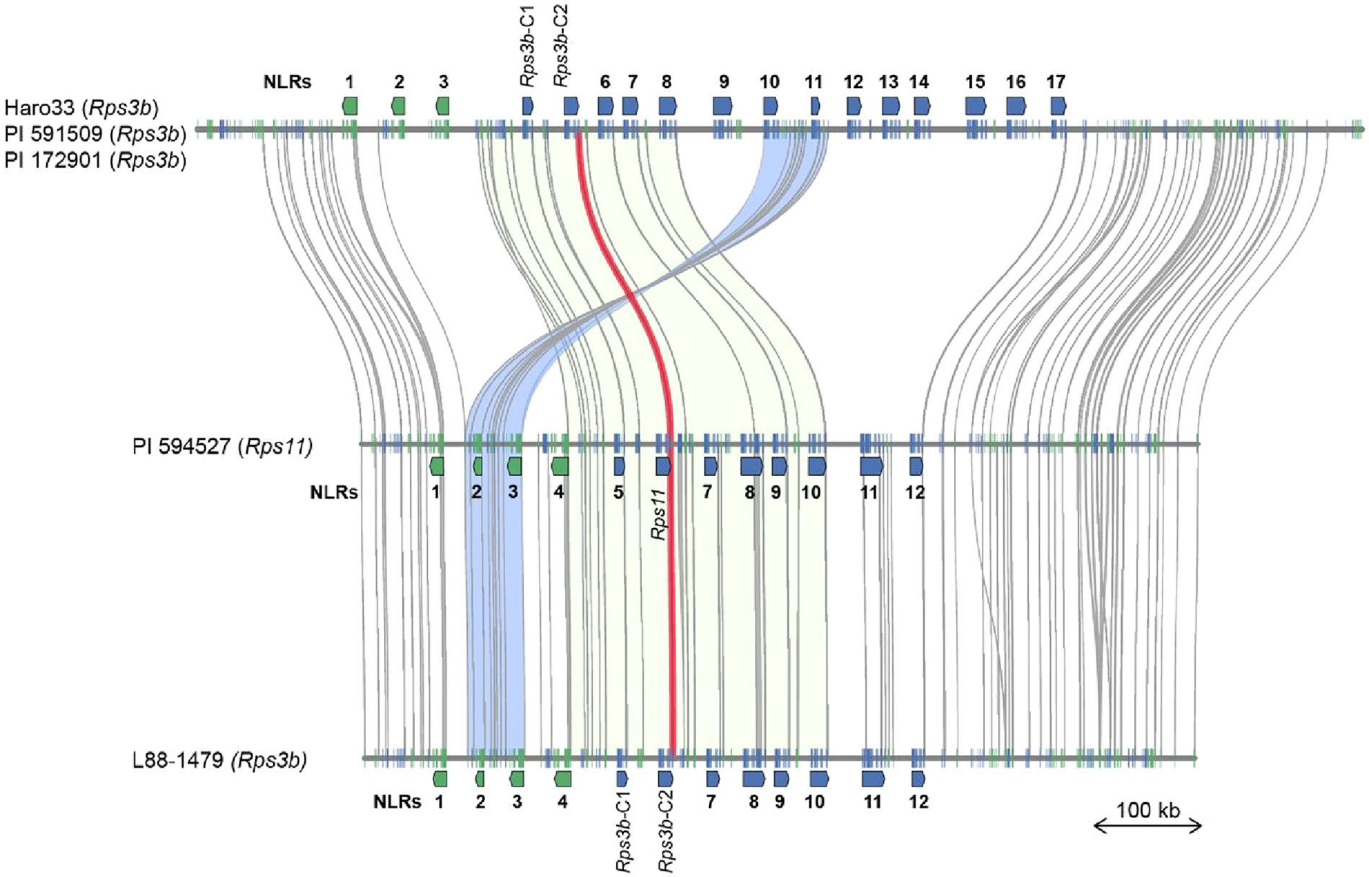
Synteny between the *Rps3b* candidate regions. Global synteny of the NLR clusters in Haro33 (*Rps3b*), L88‐1479 (*Rps3b*), and PI 594527 (*Rps11*). Green and blue lines represent the orientation of the coding DNA sequences (CDS) of all genes. The arrow boxes emphasize the nucleotide‐binding and leucine‐rich repeat (NLR) positions. Green and blue shadow areas represent conserved syntenic blocks. The red line shows the position of the *Rps3b*‐C2 and *Rps11*. PI 591509 and PI 172901 match the Haro33 haplotype in this region.

**FIGURE 4:**
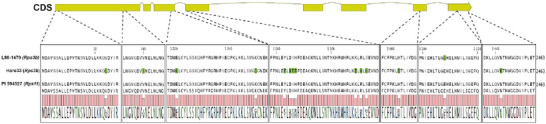
Sequence alignment of *Rps3b* candidate genes. Graphical representation of *Rps3b*‐C2 coding DNA sequences (CDS) compared to amino acid alignment of L88‐1479 (*Rps3b*), Haro33 (*Rps3b*), and PI 594527 (*Rps11*) showing localization of non‐synonymous mutations in the gene sequences.

To evaluate how or if the two *Rps3b* candidate regions were related to each other, a synteny analysis was performed. Figure 3 shows the synteny among the fully resequenced *Rps3b* lines and the equivalent region retrieved from the PI 594527 (*Rps11*) genome. In the L88‐1479 versus PI 594527 comparison, within the NLR cluster, genes from the two regions are perfectly syntenic without any structural rearrangement or variation in the gene content. Pairwise homology among the 12 NLRs ranges from 95.13% to 100%, and five of them are perfectly conserved in their amino acid sequences. In the Haro33 versus L88‐1479 comparison, we can observe two conserved blocks of genes including one 100‐kb inversion containing two NLRs. Nine NLRs from L88‐1479 have an allelic equivalent in Haro33, but only two of those are highly conserved (>99%), making them candidate genes for *Rps3b*, namely *Rps3b*‐C1 and *Rps3b*‐C2. Interestingly, *Rps11* is 99.99% identical to *Rps3b*‐C2, having only one non‐synonymous SNP over its 7392‐bp coding sequence. This same gene is also highly conserved in Haro33, with only 13 non‐synonymous SNPs present, making it 99.73% identical in its coding sequence (Figure 4). Considering that the highest homology among the 17 NLRs in Haro33 and the 12 NLRs in L88‐1479 is 89.52% and 88.29%, respectively, a homology of 99.73% can hardly be considered as random, especially regarding the highly variable nature of NLRs.

### 3.4 Phenotypes against *Avr3b* alleles

**FIGURE 5:**
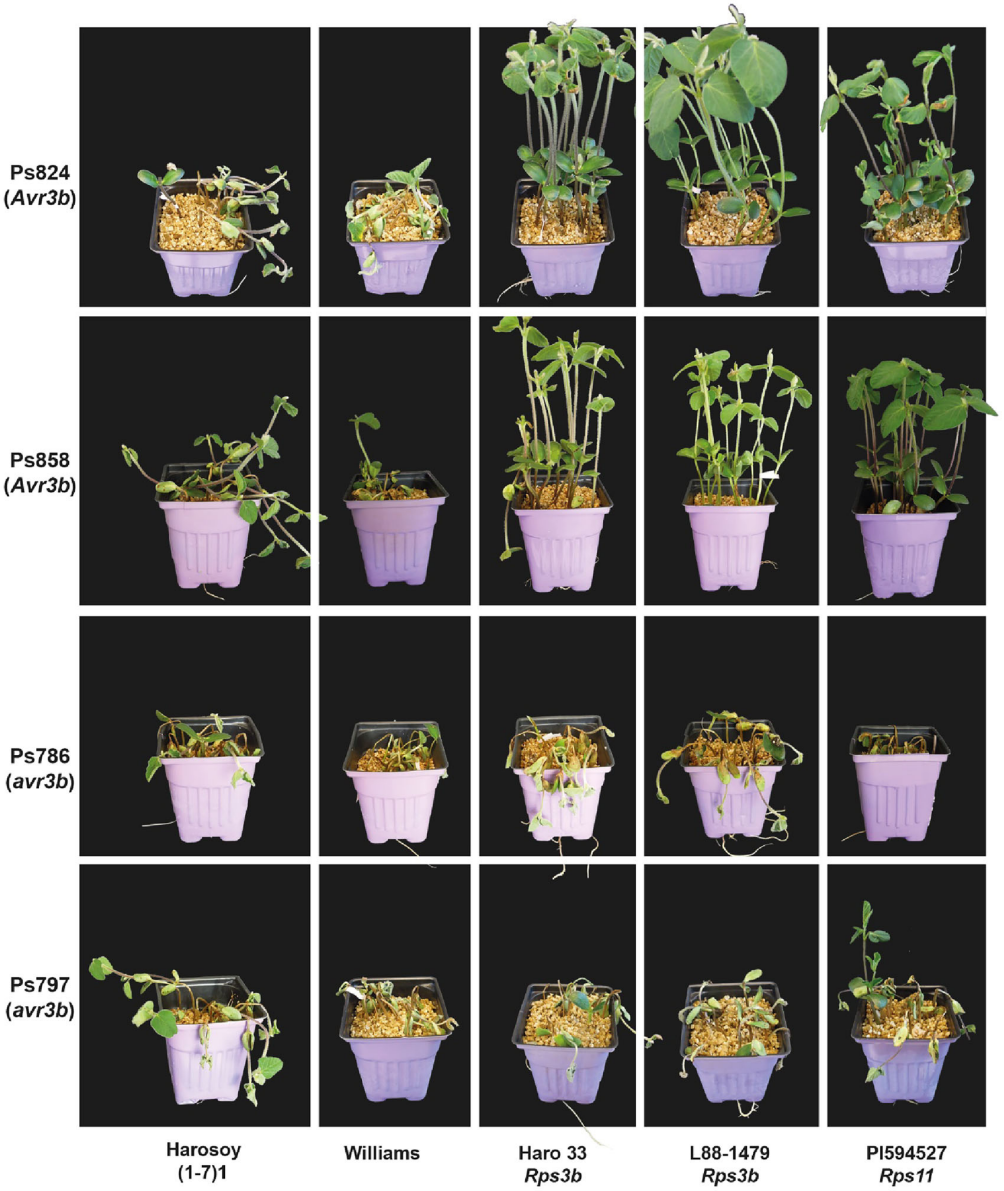
Phenotypic responses of *Rps3b* and *Rps11* lines. Phenotypes of Haro33 (*Rps3b*), L88‐1479 (*Rps3b*), PI 594527 (*Rps11*), and susceptible controls (Harosoy and Williams) using hypocotyl‐wound inoculation while confronted to *Phytophthora sojae* isolates carrying avirulent and virulent alleles of *Avr3b*.

Genotyping of our collection of *P. sojae* isolates for alleles at the *Avr3b* locus (*Avr3b/avr3b*) allowed the identification of 21 strains carrying pathotype *3b*, representing an occurrence of 8.3% over the 254 field isolates screened (Table S1). *P. sojae* isolates contrasting for their allele at the *Avr3b* locus were selected for phenotyping the resistant lines carrying *Rps3b* and *Rps11*. As shown in Figure 5, susceptible control lines displayed the expected phenotypes following inoculation. For their part, plants from all lines carrying either the allele of *Rps3b* or *Rps11* displayed a high level of susceptibility when inoculated with a strain expressing pathotype *3b*. When isolates with an avirulent allele of *Avr3b* were inoculated on the *Rps3b* and *Rps11* lines, PI 594527 (*Rps11*), L88‐1479 (*Rps3b*), and Haro33 (*Rps3b*) showed a similar resistant phenotype regardless of whether they carried *Rps3b* or *Rps11*. For all assays, no discrepancies between *Rps3b* and *Rps11* lines were observed regarding their phenotypes, showing that *Rps11* resistance can be broken by the pathotype *3b* of *P. sojae*.

## 4 DISCUSSION

### 4.1 Seeking to sort out the confusion with *Rps* genes

With some 40 *Rps* genes and alleles reported in soybean, and only two cloned and characterized for their functionality (Gao et al., 2005; Wang et al., 2021), a lot of confusion has surrounded their precise characterization, localization, potential redundancy, and practical use. In this work, we applied state‐of‐the‐art NLR gene capture combined with long‐ and short‐read sequencing technologies to precisely identify *Rps3b*, first reported 40 years ago but largely overlooked owing to its imprecise initial characterization. By comparing a panel of NILs against their respective recurrent parents, the localization and sequences of *Rps3b* alleles were specifically uncovered and, surprisingly, linked to the recently cloned *Rps11* gene reported as a new resistance gene without known allelic copies (Wang et al., 2021). Using whole‐genome resequencing and de novo assembly, we showed that four well‐studied resistant soybean accessions known to carry *Rps3b* resistance, including the line from which *Rps3b* was originally described, possess two syntenic groups of NLR genes, one with 12 and the other with 17 genes. Surprisingly, the same group of 12 genes at the same locus was also described in the publication of Wang et al. (2021) as the region hosting the putative *Rps11*.

### 4.2 *Rps3b* lies on Chr7 and not on Chr13 as previously reported

Although nomenclature suggested that *Rps3b* is allelic to *Rps3a*, a gene that was previously mapped on Chr13 (Diers et al., 1992; Ploper et al., 1985), our results indicate that *Rps3b* is harbored on an entirely different chromosome. Using a classical BSA strategy followed by genetic mapping applied to F_2:3_ families, *Rps3b* was mapped on Chr7, encompassing the region where the *Rps11* gene cluster is located. Although markers located on Chr17 were detected in the BSA, they were heterozygous in the F_2_ population and located more than 23 Mb from any other *Rps* genes, and it is conceivable that such associations were detected because of the relatively small size of the bulks (seven individuals/bulk). Consecutively to QTL mapping, only the interval on Chr7 was significantly associated with resistance in the full mapping population, ruling out markers detected on Chr3. Additionally, when markers covering the *Rps3a* region of Chr13 were evaluated in this population, no association with resistance was detected. Interestingly, QTL mapping showed what would appear to be an anomalously high recombination rate under the QTL (8 cM for a 16‐kb interval). This is most likely due to the fact that in the Williams82 reference genome, the physical size of this region is much smaller (by ∼500 kb) than it is in the lines carrying the resistant allele and that some clusters of NLR genes can be recombination hotspots (Barragan & Weigel, 2021; Choi et al., 2016). Historically, the position of *Rps3b* on Chr13 was never confirmed through linkage mapping or molecular markers. When Ploper et al. (1985) originally described this resistance gene by performing an allelism study with *Rps3a*, the authors saw no segregation of the resistance (all F_2_ families were resistant) and concluded that *Rps3a* and *Rps3b* were allelic. However, none of the *P. sojae* isolates employed by the authors carried the pathotype *3b*, which is able to infect *Rps3b*, the effector being described only a number of years later (Dong et al., 2011). Without discriminant isolates that reveal the specificity of a resistance gene, an allelism study can yield misleading results. Far from being a unique case, new resistance genes are frequently reported without being precisely identified, leading to confusion over identity and nomenclature. As an example, *Rps4* and *Rps6* were initially described as different resistance genes but are now considered either allelic or tightly linked (Sandhu et al., 2004). Another case is seen with *Rps3a* and *Rps8*, two genes co‐localizing in the same region of Chr13 and recognizing the same effector molecule, *Avr3a* (Arsenault‐Labrecque et al., 2022; Sandhu et al., 2005). Because *Rps* genes were mostly independently studied, the relationships among known genes have remained unclear. Some *Rps* loci are reported to host as many as 15 different resistance genes such as the one on Chr3 (Zhong et al., 2020). While it remains plausible that they are all unique, there is a stronger possibility of redundancy in their identification because only a minority of those genes have been associated with their corresponding effector. Thus, there is a need for comparative studies between *Rps* genes, which can be achieved by novel sequencing approaches that allow comparison among their NLR genes.

### 4.3 RenSeq as a resistance gene discovery tool

To establish a proof of concept for a new method to identify candidate genes for resistance in soybean, we chose *Rps3b*, a gene never successfully subjected to association mapping and never commercially deployed, making it a perfect scenario to apply the RenSeq method, a powerful yet still underexploited identification tool (Arora et al., 2019; Jupe et al., 2013; Witek et al., 2016). Our results regarding the prediction of a candidate region associated with resistance showed that NILs offered distinct advantages over recombinant inbred lines for this purpose. Indeed, in the case of *Rps3b*, simple NLR‐wise comparisons between recurrent parents and resistant NILs precisely and readily narrowed the search down to a 200‐kb candidate region on Chr7. To achieve a similar precision, Wang et al. (2021) had to develop and genotype an impressive mapping population of 17,050 F_4_. In this study, NLR sequences of only four plants were necessary, representing a drastic reduction in terms of time and cost. Nevertheless, developing such a population and identifying key recombinant lines have been determinant in narrowing the candidate region comprising 12 NLR genes to the one true resistance gene.

### 4.4 Are *Rps3b* and *Rps11* different alleles or different genes?

From whole‐genome resequencing of *Rps3b* resistant accessions, we have uncovered two syntenic NLR clusters on Chr7, one harboring 12 NLRs and the other harboring 17 NLRs. Haro33 and PI 591509 carried the same cluster of NLRs as PI 172901, a Turkish landrace and the original source of *Rps3b* described by Ploper et al. (1985). According to Dorrance et al. (2004), PI 172901 was the donor of resistance to PI 591509, a relationship confirmed in this study. However, the source of the gene in Haro33 was more obscure, and our results confirm that PI 172901 was also the donor of resistance. Surprisingly, L88‐1479 and Haro33, two well‐studied NILs known to carry *Rps3b*, did not share the same group of NLR genes at the resistance locus on Chr7. L88‐1479 was shown to carry a group of NLRs highly homologous to the one present in PI 594527 (*Rps11*), a Chinese landrace. But if L88‐1479 and Haro33 shared the same resistance, we surmised the source should be within genes present in the clusters. Incidentally, we have found that, within their respective genomes, they share two neighboring NLR genes, *Rps3b*‐C1 and *Rps3b*‐C2. As this family of genes is known for its tremendous diversity, such an observation is unlikely a coincidence. Because *Rps3b*‐C1 is shared with PI 594527 (*Rps11*) but not expressed according to Wang et al. (2021), *Rps3b*‐C2 remains the obvious candidate for *Rps3b*. While cloning of the gene from either source was beyond the scope of this paper, our phenotyping data confirm that both clusters confer resistance to the same *P. sojae* pathotype. From pairwise homology comparisons, we have shown that this candidate is 99.99% identical with *Rps11*, and we consider them allelic.

Even though *Rps* genes were first discovered and used in the 1960s for preventing large‐scale PRR epidemics, their proper exploitation has been hampered by the challenges associated with their precise identification. This conundrum has prevented breeders from properly assessing the efficacy of currently commercialized *Rps* genes and from introgressing new ones (Tremblay et al., 2021). While new and innovative tools now allow genotyping of effectors and detection of pathotypes in soil samples and thus enable growers to choose a soybean variety containing an effective resistance gene (R gene), knowledge of the R gene and its corresponding effector remains of paramount importance (Arsenault‐Labrecque et al., 2018; Dussault‐Benoit et al., 2020). In their report of *Rps11*, Wang et al. (2021) did not investigate the matching effector, *Avr11*, in *P. sojae* populations. As stated by the gene‐for‐gene model, every R gene recognizes a specific effector, which theoretically challenges the notion of “broad‐spectrum” resistance in the case of *Rps11*. Our results showed that *Rps3b*/*Rps11* follows the same rule, whereas *Avr3b*, an ADP‐ribose/NADH pyrophosphorylase, is recognized by the product of *Rps3b* and, by association, of *Rps11* (Dong et al., 2011). Supported by phenotyping experiments, the allele of *Avr3b* carried by the isolate determines the response of the plants to infection. Lines with *Rps3b* and *Rps11* showed the same pattern of resistance, one that coincides with the presence of the *Avr3b* allele. Since *Rps3b* was never knowingly deployed commercially, one can assume that *P. sojae* populations still largely lack the capacity to infect soybean lines carrying this gene. Incidentally, in our collection of *P. sojae* field isolates from Canada, only approximately 8% of them carried pathotype *3b*. In a similar way, this gene was reported as highly effective against *P. sojae* isolates collected in the south of Brazil, where the pathogen finds favorable conditions to cause disease. Indeed, only 19% of the isolates evaluated in the study by Costamilan et al. (2013) resulted in susceptible reactions on accessions carrying an *Rps3b* allele. Finally, Wang et al. (2021) showed that only 19.6% of the field isolates from Indiana could circumvent the resistance provided by *Rps11*, a figure similar to southern Brazil. Altogether, these field surveys showed that pathotype *3b/11* is relatively rare in *P. sojae* populations, making this gene an interesting new source of resistance for deployment in North America.

## 5 CONCLUSION

In summary, we describe here an approach to investigate NLR‐type resistance in plants. By combining RenSeq, a method specifically developed to study NLRs, with an underused population type, NILs, we were able to map *Rps3b* using only two salient genotypes. We also discovered two allelic forms for this elusive gene, and we determined that *Rps11* was a third allele. These data provide a new valuable source of resistance to *P. sojae*, one that is fully sequenced and highly effective in all major soybean‐producing regions.

## AUTHOR CONTRIBUTIONS

**Yanick Asselin**: Conceptualization; data curation; formal analysis; methodology; validation; writing—original draft. **Luann A. F. Dias**: Formal analysis. **Caroline Labbé**: Methodology; project administration; supervision. **Amandine Lebreton**: Formal analysis; methodology. **Vincent‐Thomas Boucher‐St‐Amour**: Formal analysis. **Benjamin Cinget**: Software; writing—review and editing. **François Belzile**: Supervision; writing—review and editing. **Gaspar Malone**: Formal analysis. **Francismar C. Marcelino‐Guimarães**: Conceptualization; supervision. **Richard R. Bélanger**: Conceptualization; funding acquisition; project administration; supervision; writing—review and editing.

## CONFLICT OF INTEREST STATEMENT

The authors declare no conflicts of interest.

## Supporting information




**Table S1**. Genotyping of *P. sojae* field isolates
**Table S2**. Bulked Segregant Analysis
**Table S3**. Summary table of marker segregation distortion test for an expected ratio of 1:2:1 on the F_2_ genotypes.
**Data S1**. Coding and protein sequences of *Rps3b‐*C2

## Data Availability

The datasets generated during the current study are available in the NCBI SRA repository under the bioproject PRJNA1248456.
