# Supplementary material for: Allelism of Rps3b and Rps11 revealed by NLR gene capture of resistance genes to Phytophthora sojae in soybean
Source: Plant Genome. 2025 Jun 12;18(2):e70054. doi: 10.1002/tpg2.70054 (PMC12162409; doi:10.1002/tpg2.70054)
Supplement: Supplementary file 1 — Table S1. Genotyping of P. sojae field isolates Table S2. Bulked Segregant Analysis Table S3. Summary table of marker segregation distortion test for an expected ratio of 1:2:1 on the F2 genotypes. Data S1. Coding and protein sequences of Rps3b‐C2 [file TPG2-18-e70054-s001.docx]

**Table S1.** Genotyping of *P. sojae* field isolates

| **Isolate ID** | **Harvest year** | **Avr3b genotype** | **Isolate ID** | **Harvest year** | **Avr3b genotype** |
| --- | --- | --- | --- | --- | --- |
| UL_Ps_00473 | 2018 | Avr | UL_Ps_00604 | 2018-2019 | Avr |
| UL_Ps_00474 | 2018 | Avr | UL_Ps_00605 | 2018-2019 | Avr |
| UL_Ps_00475 | 2018 | Avr | UL_Ps_00606 | 2018-2019 | Avr |
| UL_Ps_00476 | 2018 | Avr | UL_Ps_00607 | 2018-2019 | Avr |
| UL_Ps_00477 | 2018 | Avr | UL_Ps_00608 | 2018-2019 | Avr |
| UL_Ps_00478 | 2018 | Avr | UL_Ps_00609 | 2018-2019 | Avr |
| UL_Ps_00479 | 2018 | Avr | UL_Ps_00610 | 2018-2019 | Avr |
| UL_Ps_00480 | 2018 | Avr | UL_Ps_00611 | 2018-2019 | Avr |
| UL_Ps_00481 | 2018 | Avr | UL_Ps_00612 | 2018-2019 | Avr |
| UL_Ps_00482 | 2018 | Avr | UL_Ps_00613 | 2018-2019 | Avr |
| UL_Ps_00483 | 2018 | Avr | UL_Ps_00614 | 2018-2019 | vir |
| UL_Ps_00484 | 2018 | Avr | UL_Ps_00615 | 2018-2019 | Avr |
| UL_Ps_00485 | 2018 | Avr | UL_Ps_00616 | 2018-2019 | Avr |
| UL_Ps_00486 | 2018 | Avr | UL_Ps_00617 | 2018-2019 | Avr |
| UL_Ps_00487 | 2018 | Avr | UL_Ps_00618 | 2018-2019 | Avr |
| UL_Ps_00488 | 2018 | Avr | UL_Ps_00619 | 2018-2019 | Avr |
| UL_Ps_00489 | 2018 | Avr | UL_Ps_00620 | 2018-2019 | Avr |
| UL_Ps_00490 | 2018 | Avr | UL_Ps_00621 | 2018-2019 | Avr |
| UL_Ps_00491 | 2018 | Avr | UL_Ps_00623 | 2018-2019 | Avr |
| UL_Ps_00492 | 2018 | Avr | UL_Ps_00628 | 2018-2019 | Avr |
| UL_Ps_00493 | 2018 | Avr | UL_Ps_00629 | 2018-2019 | Avr |
| UL_Ps_00494 | 2018 | Avr | UL_Ps_00630 | 2018-2019 | Avr |
| UL_Ps_00495 | 2018 | Avr | UL_Ps_00631 | 2018-2019 | Avr |
| UL_Ps_00496 | 2018 | Avr | UL_Ps_00632 | 2018-2019 | Avr |
| UL_Ps_00497 | 2018 | Avr | UL_Ps_00633 | 2018-2019 | Avr |
| UL_Ps_00498 | 2018 | Avr | UL_Ps_00634 | 2018-2019 | Avr |
| UL_Ps_00499 | 2018 | Avr | UL_Ps_00635 | 2018-2019 | Avr |
| UL_Ps_00500 | 2018 | Avr | UL_Ps_00638 | 2018-2019 | Avr |
| UL_Ps_00501 | 2018 | Avr | UL_Ps_00640 | 2018-2019 | Avr |
| UL_Ps_00502 | 2018 | Avr | UL_Ps_00727 | 2019 | Avr |
| UL_Ps_00503 | 2018 | Avr | UL_Ps_00728 | 2019 | Avr |
| UL_Ps_00504 | 2018 | Avr | UL_Ps_00729 | 2019 | Avr |
| UL_Ps_00505 | 2018 | Avr | UL_Ps_00730 | 2019 | Avr |
| UL_Ps_00506 | 2018 | Avr | UL_Ps_00731 | 2019 | Avr |
| UL_Ps_00507 | 2018 | Avr | UL_Ps_00732 | 2019 | vir |
| UL_Ps_00508 | 2018 | Avr | UL_Ps_00733 | 2019 | vir |
| UL_Ps_00509 | 2018 | Avr | UL_Ps_00734 | 2019 | Avr |
| UL_Ps_00510 | 2018 | Avr | UL_Ps_00735 | 2019 | Avr |
| UL_Ps_00511 | 2018 | Avr | UL_Ps_00736 | 2019 | Avr |
| UL_Ps_00512 | 2018 | Avr | UL_Ps_00737 | 2019 | Avr |
| UL_Ps_00513 | 2018 | Avr | UL_Ps_00738 | 2019 | Avr |
| UL_Ps_00514 | 2018 | Avr | UL_Ps_00739 | 2019 | Avr |
| UL_Ps_00515 | 2018 | Avr | UL_Ps_00740 | 2019 | Avr |
| UL_Ps_00516 | 2018 | Avr | UL_Ps_00741 | 2019 | vir |
| UL_Ps_00517 | 2018 | Avr | UL_Ps_00742 | 2019 | vir |
| UL_Ps_00518 | 2018 | Avr | UL_Ps_00743 | 2019 | Avr |
| UL_Ps_00519 | 2018 | Avr | UL_Ps_00744 | 2019 | Avr |
| UL_Ps_00520 | 2018 | Avr | UL_Ps_00745 | 2019 | Avr |
| UL_Ps_00521 | 2018 | Avr | UL_Ps_00746 | 2019 | Avr |
| UL_Ps_00522 | 2018 | Avr | UL_Ps_00747 | 2019 | Avr |
| UL_Ps_00523 | 2018 | Avr | UL_Ps_00748 | 2019 | Avr |
| UL_Ps_00524 | 2018 | Avr | UL_Ps_00749 | 2019 | Avr |
| UL_Ps_00525 | 2018 | Avr | UL_Ps_00750 | 2019 | Avr |
| UL_Ps_00526 | 2018 | Avr | UL_Ps_00751 | 2019 | vir |
| UL_Ps_00527 | 2018 | Avr | UL_Ps_00752 | 2019 | vir |
| UL_Ps_00528 | 2018 | Avr | UL_Ps_00753 | 2019 | Avr |
| UL_Ps_00529 | 2018 | Avr | UL_Ps_00754 | 2019 | Avr |
| UL_Ps_00530 | 2018 | Avr | UL_Ps_00755 | 2019 | Avr |
| UL_Ps_00531 | 2018 | Avr | UL_Ps_00756 | 2019 | Avr |
| UL_Ps_00532 | 2018 | Avr | UL_Ps_00757 | 2019 | Avr |
| UL_Ps_00533 | 2018 | Avr | UL_Ps_00758 | 2019 | Avr |
| UL_Ps_00534 | 2018 | Avr | UL_Ps_00759 | 2019 | Avr |
| UL_Ps_00535 | 2018 | Avr | UL_Ps_00760 | 2019 | Avr |
| UL_Ps_00536 | 2018 | Avr | UL_Ps_00761 | 2019 | Avr |
| UL_Ps_00537 | 2018 | Avr | UL_Ps_00762 | 2019 | Avr |
| UL_Ps_00538 | 2018 | Avr | UL_Ps_00763 | 2019 | Avr |
| UL_Ps_00539 | 2018 | Avr | UL_Ps_00764 | 2019 | Avr |
| UL_Ps_00540 | 2018 | Avr | UL_Ps_00765 | 2019 | Avr |
| UL_Ps_00541 | 2018 | Avr | UL_Ps_00766 | 2019 | Avr |
| UL_Ps_00542 | 2018 | Avr | UL_Ps_00767 | 2019 | Avr |
| UL_Ps_00543 | 2018 | Avr | UL_Ps_00768 | 2019 | vir |
| UL_Ps_00544 | 2018 | Avr | UL_Ps_00769 | 2019 | vir |
| UL_Ps_00545 | 2018 | Avr | UL_Ps_00770 | 2019 | Avr |
| UL_Ps_00546 | 2018 | Avr | UL_Ps_00772 | 2019 | Avr |
| UL_Ps_00547 | 2018 | Avr | UL_Ps_00773 | 2019 | vir |
| UL_Ps_00548 | 2018 | Avr | UL_Ps_00774 | 2019 | vir |
| UL_Ps_00549 | 2018 | Avr | UL_Ps_00775 | 2019 | Avr |
| UL_Ps_00550 | 2018 | Avr | UL_Ps_00776 | 2019 | Avr |
| UL_Ps_00551 | 2018 | Avr | UL_Ps_00777 | 2019 | Avr |
| UL_Ps_00552 | 2018 | Avr | UL_Ps_00778 | 2019 | Avr |
| UL_Ps_00553 | 2018 | Avr | UL_Ps_00779 | 2019 | vir |
| UL_Ps_00554 | 2018 | Avr | UL_Ps_00780 | 2019 | Avr |
| UL_Ps_00555 | 2018 | vir | UL_Ps_00781 | 2019 | Avr |
| UL_Ps_00556 | 2018 | Avr | UL_Ps_00782 | 2019 | Avr |
| UL_Ps_00557 | 2018 | Avr | UL_Ps_00783 | 2019 | Avr |
| UL_Ps_00559 | 2018 | Avr | UL_Ps_00784 | 2019 | Avr |
| UL_Ps_00560 | 2018 | Avr | UL_Ps_00785 | 2019 | Avr |
| UL_Ps_00561 | 2018 | Avr | UL_Ps_00786 | 2019 | vir |
| UL_Ps_00562 | 2018 | vir | UL_Ps_00787 | 2019 | Avr |
| UL_Ps_00563 | 2018 | Avr | UL_Ps_00788 | 2019 | vir |
| UL_Ps_00564 | 2018 | Avr | UL_Ps_00789 | 2019 | Avr |
| UL_Ps_00565 | 2018 | Avr | UL_Ps_00790 | 2019 | Avr |
| UL_Ps_00566 | 2018 | Avr | UL_Ps_00791 | 2019 | Avr |
| UL_Ps_00567 | 2018 | Avr | UL_Ps_00792 | 2019 | vir |
| UL_Ps_00568 | 2018 | Avr | UL_Ps_00793 | 2019 | Avr |
| UL_Ps_00569 | 2018 | Avr | UL_Ps_00794 | 2019 | Avr |
| UL_Ps_00570 | 2018 | Avr | UL_Ps_00795 | 2019 | Avr |
| UL_Ps_00571 | 2018 | Avr | UL_Ps_00796 | 2019 | Avr |
| UL_Ps_00572 | 2018 | Avr | UL_Ps_00797 | 2019 | vir |
| UL_Ps_00573 | 2018 | Avr | UL_Ps_00798 | 2019 | Avr |
| UL_Ps_00574 | 2018 | Avr | UL_Ps_00799 | 2019 | Avr |
| UL_Ps_00575 | 2018 | Avr | UL_Ps_00800 | 2019 | Avr |
| UL_Ps_00576 | 2018 | Avr | UL_Ps_00801 | 2019 | Avr |
| UL_Ps_00577 | 2018 | vir | UL_Ps_00802 | 2019 | Avr |
| UL_Ps_00578 | 2018 | Avr | UL_Ps_00803 | 2020 | Avr |
| UL_Ps_00579 | 2018 | Avr | UL_Ps_00804 | 2020 | Avr |
| UL_Ps_00581 | 2018 | Avr | UL_Ps_00805 | 2020 | Avr |
| UL_Ps_00582 | 2018 | Avr | UL_Ps_00806 | 2020 | Avr |
| UL_Ps_00583 | 2018 | Avr | UL_Ps_00807 | 2020 | Avr |
| UL_Ps_00584 | 2018 | Avr | UL_PS_00814 | 2020 | Avr |
| UL_Ps_00585 | 2018 | Avr | UL_PS_00815 | 2020 | Avr |
| UL_Ps_00586 | 2018 | Avr | UL_PS_00816 | 2020 | Avr |
| UL_Ps_00587 | 2018 | Avr | UL_PS_00817 | 2020 | Avr |
| UL_Ps_00588 | 2018 | Avr | UL_PS_00818 | 2020 | Avr |
| UL_Ps_00589 | 2018 | Avr | UL_PS_00819 | 2020 | Avr |
| UL_Ps_00590 | 2018 | Avr | UL_PS_00820 | 2020 | vir |
| UL_Ps_00591 | 2018 | Avr | UL_PS_00822 | 2020 | Avr |
| UL_Ps_00592 | 2018 | Avr | UL_PS_00823 | 2020 | Avr |
| UL_Ps_00593 | 2018 | Avr | UL_PS_00824 | 2020 | Avr |
| UL_Ps_00595 | 2018 | Avr | UL_PS_00825 | 2020 | Avr |
| UL_Ps_00596 | 2018 | Avr | UL_PS_00826 | 2020 | Avr |
| UL_Ps_00597 | 2018 | Avr | UL_PS_00829 | 2019 | Avr |
| UL_Ps_00598 | 2018 | vir | UL_PS_00830 | 2019 | Avr |
| UL_Ps_00599 | 2018 | Avr | UL_PS_00831 | 2019 | Avr |
| UL_Ps_00600 | 2018 | Avr | UL_PS_00832 | 2019 | Avr |
| UL_Ps_00601 | 2018 | Avr | UL_PS_00857 | 2020 | Avr |
| UL_Ps_00603 | 2018-2019 | Avr | UL_PS_00858 | 2020 | Avr |

**Table S2.** Bulked Segregant Analysis revealed six SNPS with full association between bulks’ phenotypes and genotypes and one SNP with partial association. Chr: soybean chromosome; Map: map position in the reference genome Williams82_v2; “./.”; undetermined.

|  |  |  | **OBSERVED GENOTYPES** | | | | | |  |
| --- | --- | --- | --- | --- | --- | --- | --- | --- | --- |
| **Chr** | **Map** | **Marker type** | **PI**  **591509** | **BRS**  **268** | **R**  **Bulk1** | **R**  **Bulk2** | **S**  **Bulk1** | **S**  **Bulk2** | **Close *Rps* loci** |
| 3 | 2,971,986 | InDel | ./. | C/C | ./. | ./. | C/C | C/C | *Rps1, Rps7, Rps9, RpsUN1, RpsYu25, RpsWY, RpsQ, RpsYD29, RpsX, RpsYD25* |
| 7 | 6,463,180 | SNP | A/A | G/G | A/A | A/A | G/G | A/G | *Rps11* |
| 7 | 8,028,697 | SNP | T/T | A/A | T/T | T/T | A/A | **./.** |  |
| 7 | 9,233,014 | SNP | A/A | G/G | A/A | A/A | G/G | A/G |  |
| 17 | 7,744,589 | SNP | G/G | A/A | G/A | G/A | A/A | A/A |  |
| 17 | 7,780,377 | SNP | T/T | C/C | T/C | T/C | C/C | C/C |  |

**Table S3.** Summary table of marker segregation distortion test for an expected ratio of 1:2:1 on the F2 genotypes. Markers were named according to their physical coordinates in chromosome 3, 7 and 13 of the soybean genomic reference Glyma.Wm82.a2.v1 (Soybase.org). Marker type indicated if a marker comes from targeted GBS (tGBS) or Kompetitive Allele Specific PCR (KASP). The following columns indicate, AA or A: the number of genotypes same as the first parent (P1); AB: the number of genotypes same as F1; BB: the number of genotypes same as the second parent (P2); Missing (%): the number of missing genotypes; ChiSquare: χ^2^-test statistics testing for segregation distortion of markers with the expected ratio of 1:2:1; P-Value: the corresponding probability for the χ^2^-test statistics, i.e., which is equal to P(x> ChiSquare) as generated by ICIM analysis from IciMapping v4.2 for segregation distortion of markers used in this study.

|  | |  | Observed Frequency | | |  |  |  |  |  |
| --- | --- | --- | --- | --- | --- | --- | --- | --- | --- | --- |
| Marker name | Marker Type | | AA or A_ | AB | BB | Total | Missing(%) | ChiSquare | Pr>ChiSq | Deleted |
| Chr3_3000693 | | tGBS | 36 | 72 | 56 | 164 | 1.8 | 7.32** | 0.026 | Yes |
| Chr3_3007088 | | tGBS | 36 | 75 | 56 | 167 | 0 | 6.52** | 0.038 | No |
| Chr3_3022388 | | tGBS | 36 | 75 | 56 | 167 | 0 | 6.52** | 0.038 | Yes |
| Chr3_3068301 | | tGBS | 36 | 75 | 56 | 167 | 0 | 6.52** | 0.038 | Yes |
| Chr3_3071140 | | tGBS | 36 | 74 | 56 | 166 | 0.6 | 6.77** | 0.034 | Yes |
| Chr3_3115149 | | tGBS | 36 | 49 | 56 | 141 | 15.57 | 18.79** | 0 | Yes |
| Chr3_3115739 | | tGBS | 30 | 44 | 39 | 113 | 32.34 | 6.96** | 0.031 | Yes |
| Chr3_3195054 | | tGBS | 35 | 76 | 55 | 166 | 0.6 | 6** | 0.05 | No |
| Chr3_3576761 | | tGBS | 33 | 78 | 56 | 167 | 0 | 7.06** | 0.029 | No |
| Chr3_3617955 | | tGBS | 33 | 78 | 56 | 167 | 0 | 7.06** | 0.029 | Yes |
| Chr3_3992330 | | tGBS | 35 | 74 | 58 | 167 | 0 | 8.5** | 0.014 | No |
| Chr7_5018178 | | tGBS | 40 | 87 | 40 | 167 | 0 | 0.29^NS^ | 0.864 | No |
| Chr7_5082788 | | tGBS | 40 | 87 | 40 | 167 | 0 | 0.29^NS^ | 0.864 | Yes |
| Chr7_5085175 | | tGBS | 129 | 0 | 38 | 167 | 0 | 0.45^NS^ | 0.503 | No |
| Chr7_5089853 | | tGBS | 131 | 0 | 34 | 165 | 1.2 | 1.7^NS^ | 0.192 | No |
| Chr7_5114882 | | tGBS | 39 | 88 | 40 | 167 | 0 | 0.5^NS^ | 0.78 | No |
| Chr7_5166637 | | KASP | 29 | 73 | 30 | 132 | 20.96 | 1.5 ^NS^ | 0.472 | Yes |
| Chr7_5246639 | | KASP | 31 | 41 | 36 | 108 | 35.33 | 6.72** | 0.035 | Yes |
| Chr7_5265136 | | tGBS | 39 | 74 | 42 | 155 | 7.19 | 0.43 ^NS^ | 0.806 | Yes |
| Chr7_5268818 | | tGBS | 40 | 85 | 42 | 167 | 0 | 0.1^NS^ | 0.95 | No |
| Chr7_5276183 | | KASP | 40 | 85 | 42 | 167 | 0 | 0.1^NS^ | 0.95 | Yes |
| Chr7_5320507 | | tGBS | 40 | 83 | 42 | 165 | 1.2 | 0.05^NS^ | 0.973 | Yes |
| Chr7_5361843 | | KASP | 24 | 59 | 31 | 114 | 31.74 | 1 ^NS^ | 0.607 | Yes |
| Chr7_5385055 | | KASP | 40 | 85 | 42 | 167 | 0 | 0.1^NS^ | 0.95 | Yes |
| Chr7_5391104 | | tGBS | 40 | 86 | 41 | 167 | 0 | 0.16^NS^ | 0.922 | No |
| Chr7_5411244 | | tGBS | 40 | 86 | 41 | 167 | 0 | 0.16^NS^ | 0.922 | Yes |
| Chr7_5456442 | | tGBS | 40 | 86 | 41 | 167 | 0 | 0.16^NS^ | 0.922 | Yes |
| Chr7_5482581 | | tGBS | 39 | 72 | 42 | 153 | 8.38 | 0.65 ^NS^ | 0.724 | No |
| Chr7_5498675 | | KASP | 37 | 63 | 39 | 139 | 16.77 | 1.27 ^NS^ | 0.529 | No |
| Chr7_6144424 | | KASP | 38 | 69 | 45 | 152 | 8.98 | 1.93 ^NS^ | 0.38 | No |
| Chr13_28128626 | | KASP | 34 | 92 | 36 | 162 | 2.99 | 3.04^NS^ | 0.219 | No |
| Chr13_28544205 | | KASP | 21 | 55 | 15 | 91 | 45.51 | 4.76* | 0.093 | Yes |
| Chr13_28928028 | | KASP | 36 | 88 | 37 | 161 | 3.59 | 1.41^NS^ | 0.494 | No |
| Chr13_29166737 | | KASP | 36 | 77 | 39 | 152 | 8.98 | 0.14 ^NS^ | 0.93 | No |
| Chr13_29395603 | | KASP | 36 | 87 | 37 | 160 | 4.19 | 1.24 ^NS^ | 0.539 | No |
| Chr13_29909569 | | KASP | 23 | 58 | 20 | 101 | 39.52 | 2.41 ^NS^ | 0.3 | Yes |
| Chr13_30028197 | | KASP | 33 | 88 | 36 | 157 | 5.99 | 2.41 ^NS^ | 0.299 | No |
| Chr13_30502974 | | KASP | 37 | 77 | 36 | 150 | 10.18 | 0.12 ^NS^ | 0.942 | No |
| Chr13_30644467 | | KASP | 38 | 87 | 37 | 162 | 2.99 | 0.9^NS^ | 0.637 | No |
| Chr13_30792409 | | KASP | 39 | 86 | 36 | 161 | 3.59 | 0.86^NS^ | 0.649 | No |
| Chr13_30792474 | | KASP | 28 | 73 | 14 | 115 | 31.14 | 11.77** | 0.003 | Yes |
| Chr13_30853462 | | KASP | 125 | 0 | 24 | 149 | 10.78 | 6.28** | 0.012 | No |
| Chr13_30865023 | | KASP | 27 | 59 | 17 | 103 | 38.32 | 4.13* | 0.127 | Yes |
| Chr13_30940893 | | KASP | 38 | 83 | 37 | 158 | 5.39 | 0.42 ^NS^ | 0.812 | No |

**Data S1.** Coding and protein sequences of *Rps3b-*C2

L88-1479 *Rps3b* coding sequence

>ATGGACGCTGTGTCATCCGCACTACTAGAGCCAGTAACTAATTCTGTGTTGGATCTGCTTAAAAAGCAAGTGGATTACATCCGTTACAGGCGAAACTTTGATGAACTAGACGAGTGTGTTAAGCAGCTTAAACATAAAAAGGAGATAGTAGATCATCAATGTGAGGAAGCTGTCAAAAATGGACACGAAATTGAAGGTAAGGTTAGAGAATGGTTAGGGAAAGTGGGTAAATTTGAGACAGAAGTGGAGAAGTATTGGAACGATGATGGCCACAAAAAGACACGGTTTTCCAACTATTTATTTCCTTACTTTAGGCATAGACTAGGCAGACTAGCAAAGAAGATGGCAGTTGAGGGTAAAAAGATAACCGATGATTGCCCAAAGTCTGATGAAATTGCCTATAGGGTATACGTAACATCTAATTATGCCATTTTGTCTAATAATGACCTTATGGATTTTGGTTCTAGAAAATCCATAATGGAACAAATAATGGCAACACTTGTTGAAGATCCCACTGTGAAAATGATTGGAGTGTATGGACGAAGTGGGGTGGGTAAGAGCACTTTAATCAAAGCAATTGCTAAAATTGCTCGAGACAAGAAGTTGTTTAATGTGGTGGCTTTTTCAGAAATAACAGACAACCCCAATCTAAAACAAGTCCAGGAAGATATTGCTTACCCTTTGGGATTGAAATTGGAAGGAGAAGGTGAGAATGTAAGAGCTGATCATCTACGAAGGAGGTTAAAGAAAGAGAAAGAGAACACCCTTATAATCTTGGATGACCTTTGGGACAGATTAGACTTGAATAGGTTGGGAATTCCACTTGATGGTGATGTTGATGATAAACAGGGTCCCAAAGGGCCGACAAAAGAAAAATCTCTTGCTGATTATAAGGGTTGCAAAATTTTGCTAACTTCAAGGAAACGAAATGTATTAACGGATAAAATGGAAGTTAAATTAACTTTCTGTGTAGAGGAATTAGATGAAAAAGATGCTCTGAAGTTGTTTCGGAAGGAGGCTGCAATACAAGGTGAAATGTCCAAGTCTAAAAAAGAAATTGTTAAGAAGTATTGTGCTGGGTTACCTATGGCAATAGTTACAGTTGGAAGGGCATTAAGAGACAAGAGCGACTCAGAGTGGGAAAAACTTAAAAACCAAGAACTGGTGGGAGTTCAGAATCCGATGGAGATTTCTGTAAAAATGAGTTATGACCATCTAGAAAATGAGGAGCTCAAGTCCATTTTCTTTCTTTGTGCTCAAATGGGTCATCAACCCCTAATTATGGACTTGGTGAAGTATTGCTTTGGTTTGGGAATACTTGAAGGGGTCTACTCGCTTGGGGAAGCTCGGGACAGAATATCTACATCAATCAAAAAGCTGAAAGACTCAGGTTTGGTGTTGGATGGAAGTTCTAGTATTCATTTCAATATGCACGATCTGGTTCGAGATGCTGCTTTATCTATAGCACAGAACGAGCAAAATGTATTTACTTTGAGAAATGGGAAACTTAATGATTGGCCTGAACTCAAGAGGTGCACTTCTATTTCTATATGCAATAGTGATATCATTGATGAGCTTCCTAATGTTATGAACTGTCCTCAACTTAAATTTTTCCAAATTGACAATGATGATCCATCTTTAAAAATACCTGAGAGTTTTTTTAAGAGAATGAAAAAACTCAGAGTGTTAATATTGACTGGCTTTCATCTATCAAGCTTACCATCATCAATTAAGTGCCTATCAGACCTCAGATTGCTTTGTTTGGAGCGATGCACTTTAGATCACAACTTATCCATCATAGGGAAGCTGAAAAAATTAAGAATTCTCAGCTTTTCTGGATCTCGAATTGAAAATTTGCCAGCTGAGTTGAAGGACTTGGATAAACTACAATTACTAGACATCAGCAATTGTTCAATAGTCACCATGATTCCACCTAATCTTATATCAAGGTTGACTTCGTTGGAAGAGCTGTATGTAAGAAAGTGTTTCATGGAAGTGTCGGAGGAAGGAGAGAGAAACCAAAGTCAAAATTCATTTATTTCTGAACTAAAGCATTTGCATCAATTGCAAGTGGTGGACTTAAGCATTCCATGTGCTGAATTTTTTGCAAAGGAATTGTTCTTTGACAACTTAAGTGATTACAAGATTGAGATTGGGAACTTCAAAACTCTTTCAGCTGGAGATTTCAGAATGCCTAATAAGTATGAAAATTTCAAATCTTTGGCATTGGAGCTGAAGGATGACACTGACAATATTCACTCTCAGACAGGAATAAAGTTGTTGTTTGAAACAGTTGAAAATTTGTTGTTGGGAGAGCTGAATGGTGTTCAAGATGTTGTTAATGAGTTGAATTTGAATGGATTTCCACATCTGAAACACTTTTCCATCGTAAACAACCCTAGCATCAAATATATCATCAACTCAAAGGATTTGTTTTATCCTCAGGATGTTTTTCCCAAGTTGGAATCTCTATGCCTCTACAAACTAAAAGAGATAGAGATGATATACTTTAGTTCAGGTACAGAGATGATATGCTTTAGTCCATTTACAGATTGCTCATTCACCAAATTAAAAACCATCAAGGTCGAGAAGTGTGATCAATTGAAGAATCTTTTCTCCTTTTGCATGGTTAAATTGCTTGCGAGTCTTGAAACAATTGGTGTTTCCAATTGTGGTTCTTTAGAGGAGATCATTAAAATACCAGACAATTCTGATAAGATTGAGTTTCTTAAGTTGATGTCTTTGTCACTTGAATCATTATCATCATTCACTAGTTTTTATACCACAGTAGAGGGGTCTTCTACAAACAGAGATCAGATACAAATTACTGTTATGACTCCTCCTCTTTTTGGTGAACTGGTTGAAATACCAAACTTAGAGAACTTGAATTTAATCTCAATGAACAAGATCCAGAAGATATGGAGCGACCAGCCCCCGTCAAACTTCTGCTTTCAGAACTTAATAAAATTAGTTGTGAAAGATTGTCAAAATTTGAGATATTTGTGTTCATTGTCCGTGGCCAGCAGTTTGAGGAAACTGAAAGGCCTCTTTGTAAGCAACTGTAAAATGATGGAGAAGATTTTTAGCACAGAAGGAAATAGTGCAGACAAGGTTTGCGTCTTTCCTAAGTTGGAGGAAATTCACCTCGACCAAATGGATGAGTTAACAGACATATGGCAAGCTGAAGTGAGTGCTGATTCCTTTTCTAGTCTCACTTCTGTGAACATTCGTAGTTGTCATAAACTAGACAAAATTTTTCCGAGTCACATGGAAGGATGGTTTGCGAGTTTGAACAGCTTGAAGGTTTCTTTTTGTGAGTCAGTGGAAGTGATTTTTGAAATCAAAGATTCTCAGCAAGTAGATGCATCTGGTGGGATAGACACAAATTTGCAGGTTGTTTATGTAAGTGAACTCCCAAAGTTGGAGCAGGTGTGGAGCAGGGATCCAGGAGGAATTCTTAACTTCAAAAAACTGCAGAGTATAGAGATGGATGATTGTGAAAGACTGAGGAATGTATTTCCAGCTTCTGTGGGCAAAGATGTTCCAAAGCTTGAATACATGTCGGTCATAGAGTGTGATGGAATTGTGGAAATTGTTGCCTGTGAAGATGGATCCGAAACAAACACTGAACAATTAGTGTTTCCTGAACTAACCGACATGGAATTATGTTACCTATCAAGCATCCAGCATTTCTACAGGGGGAGACATCCTATAGAGTGTCCAAAATTGAAGAAGTTGTCAGTAGGGAAATGTAACGAGAAGCTAAAAACATTCGGAACCGGAGAAAGGAGCAATGAAGAAGATGAAGCAGTTATGTCAGCTGAAAAGATATTCCCCAACTTGGAGTATTTGGATATTCACTTTGACGAAGCACAGAAGTGGTTATTGAGCAACACTGTGAAGCATCGAATGCACCGTTTAAAAAAGCTTAGGTTAAGCGAAGTTAATGATGGTGAACGTCTCTGTCAAATTCTGTACAGAATGCCAAATCTAGAAAAGTTATACTTGCCGATGGCTAAACATTTGCTTAAAGAGTCGTCGGAGTCCCGTTTGGGAACCCTATTACAGCTGAAGGAATTGGATTTGTGGAGGTCGGAGATAAAGGATATAGGATTTGAACGAGAACCAGTTCTACAGAGACTAGAGCTTTTGAGCTTATTTAAGTGCCATAAATTGAGGAATTTGGGTCCTCCCTCGGTATCATTGGCTTACTTGACAAATTTGAAAGTAGAGTATTGTTATGGATTAAGGAATTTAATGGCATCCTCAACGGCAAAAAGCTTGGTTCAACTTAAGTCCATGAAGATAAGAAAATGTTGTAAATTAGAGGAAATAGTAAGCGATGAGGGAAATGAAGAAGAAGAGCAAATAGTGTTTGGCAAATTGATTACTATAGAACTTGAGGGGCTAAAAAAGCTGAAAAGTTTTTGCAGTTCCAAGAACTATGAATTCAAATTCCCGTCATTGGAAGGATTGATTGTGAGAAAATGCCCAATGATGCATACATTCACGGAGGGTGACGCAAGAGCACCAAAGTTAGAAAACACAGTTACTGCTAAAGAAGAAGGAAAAGAGGAAGCCAAATGGCAGTGGGAAGGAGACTTGAATTCCACCATACAAAAAGGTTTCAACAAGCTTTTGGAGTCTGCAAGTACTGAATCATCTCTTAGTCTCATAGATAGCCCACTACAAGTGATATGGCTTGACTCACGGCGGATCCCAAAGTCGTGCTTCAGTAACTTGACGGAATTGACTGTGCACGGATGCCAATTTTTAACAGATGTTGTCATACCCTTCTATTTACTTCCTTTCTTAACTAATTTGCAACAATTACAAGTCTCGGACTGTCGTTCTGTGAAAAGCATATTTGACGTGAAAACAGCTATGGGATTGGGAGCAGCAGCCTTCCCTAGACCTCTCCCTTTTTCCCTCAAGAAATTGACTTTAGAGTGGCTGCCAAAACTGGAGAATGTCTGGAATGAAGATCCTCATGGAATTCTAACCATGCAACTTCTACAACATGTAAAGGTTAAAAATTGTAAATGCCTTACAAGTGTGTTTCCGGCATCATTAGCCAAAGATCTTGAAAAACTAGTTGTCAAAGACTGTGAGGGATTGATAGAAATTGTTGCAGAGGATAATGCAGATCCAAGAGAAGCAAATCTGGAGCTTACGTTCCCTTGTCCCTGTGTGAGCTCATTGAAACTACAAGGTTTGCCCAAGTTCAAGTATTTTTACTACTGCTCACTGCAGTGTGACATGTTCCAGACACCTACCAAGGATGAAATGCCTACATCCAACTTACAGTGCCTGTCACTCGGTGAAAAAGGACTGGAGATGATCAAGCGTGCAGAATTTCAGAGAAACTTCTTACACAAGTTACAAGTTCTTACTCTGTGCTTTCATATTGGGTCGAATGTATTTCCATATGAAATTCTACAACTGGCGCCCAATATAGAGAAGCTTGTGGTGTGTGATGGTTCCTTCAAGGAGATTTTCTGCTTTGATAGTCTTAATGTGGATGAGGCTGGACTCCTATTACAGCTCAAAGTCTTATGCTTGGAGTCCCTTCCAGAGCTTGTTTCCATTGGGTTAGAGAACTCTTGGATTCAGCCCTTACTGGGAAATCTAGAAACCTTGGAAGTAATAGGTTGTTCTAGTTTAAAAGACTTGGTACCATCTACAGTGTCTTTTTCCAATCTGACATATTTGGAAGTAGAAAGATGCCATTGCCTGCTATATTTGTTCACATCCTCCACAGCAAGAAGTTTGGGTCAACTCAAAAGAATGGAGATAAAATGGTGTGGTTCAATTGAAGAGGTAGTAGTCTCTAAGGAGGGGGATGAATCACATGAGGAGGAGATTATATTTCCGCAGCTCAATTGTTTGAAACTTGAATATTTATCAAAGCTGAGAAGCTTCTATAAAGGAAGTTTATTAAGTTTCCCATCATTGGAGGAATTGTCAGTAATCCGTTGCGAGTGGATGGAAACATTATGTCCAGGTACCCTTAAAGCAGACAAGTTGGTTCAAGTTCAACTTAAGGAGAGTTATTCTTGGAGGCACTCAGATCCTATCAAATTGGAAAATGACCTGAACTCTACCATGCGGGAGGCATTTTGGAAAAAGTTATGGAAGTCTGCAGATACGGAATTCAGTATTGACCTCAAAGATAGCCCAGTACAAGAGATATGGCTTAGGCTTCACTCACTGCATATCCCCCCACACTTCTGCTTCCCTAAGTTACACACCTTGATTGTGGACGGCTGCCATTTTTTATCAGATGCGGTCTTACCCTTCTCTTTACTTCCTTTATTACCTAATTTGAAAACATTGGAAGTTCGAAACTGTGATTTTGTGAAAATCATATTTGATGTGACAACTATGGGACCACTCCCTTTTGCCCTCAAGACATTGGCACTGTGTGATCTGCCAAATCTGGAGAATGTTTGGAATTCAAATGTTGAGCTTACGTTCCCCCAAGTCAAGTCATTGGCACTCTGTGATCTGCCAAAGTTAAAGTATGACATCTTGAAGCCATTTACACATCTAGAACCACATACTCTAAATCAAGTCTGTATTCAAAAGCTTACACCCAACATAGAGCACCTGACACTCGGTCAACATGAACTCAACATGATTTTGAGTGGAGAATTCCAGGGAAACCACTTAAACGAGTTAAAAGTGCTTGCTCTGTTCTTTCATATTGAATCCGATGTATTTCTACAACGGGTGCCCAATATAGAGAAGCTTGAGGTGCGTGATGGTTCCTTCAAAGAGATTTTCTGCTTTGATAGCCTTAATGTGGATGAGGATGGATTGGTTTCACAGCTGAAAGTGATATGCCCGGACTCCCTTCCAGAGCTTGTTTCCATTGGGTCAGAGAACTCTGGGATTGTGCCCTTTCTCAGAAATCTAGAAACATTGCAAGTAATCAGCTGTTTCAGTTCAATAAATCTGGTACCATGCACAGTGTCTTTTTCCAATCTGACATATTTGAAAGTAGAAAGTTGCAAGAGTCTGCTATATTTGTTCACATCCTCAACAGCAAGAAGTTTGGGTCAACTCAAAACAATGGAGATAAGTTGGTGTAATTCAATTGAAGAGATAGTGTCTTCAACAGAGGAAGGGGATGAATCAGATGAGAATGAGATAATATTTCAGCAGCTCAATTGTTTGAAACTTGAATTTTTATTTAAGCTGAGAAGGTTCTACAAAGGGAGTTTAAGTTTCCCGTCCTTGGAGGAATTCACAGTATGGCGTTGCGAGAGGATGGAAAGTTTGTGTGCAGGTACAGTCAAAACAGACAAGCTGTTACAAGTGAATACTAATTGGGGCGGAGATGTTATCCCATTGGAAACTGATCTGAACTCTGCCATGCAAAACCGATAG

L88-1479 *Rps3b* protein sequence

>MDAVSSALLEPVTNSVLDLLKKQVDYIRYRRNFDELDECVKQLKHKKEIVDHQCEEAVKNGHEIEGKVREWLGKVGKFETEVEKYWNDDGHKKTRFSNYLFPYFRHRLGRLAKKMAVEGKKITDDCPKSDEIAYRVYVTSNYAILSNNDLMDFGSRKSIMEQIMATLVEDPTVKMIGVYGRSGVGKSTLIKAIAKIARDKKLFNVVAFSEITDNPNLKQVQEDIAYPLGLKLEGEGENVRADHLRRRLKKEKENTLIILDDLWDRLDLNRLGIPLDGDVDDKQGPKGPTKEKSLADYKGCKILLTSRKRNVLTDKMEVKLTFCVEELDEKDALKLFRKEAAIQGEMSKSKKEIVKKYCAGLPMAIVTVGRALRDKSDSEWEKLKNQELVGVQNPMEISVKMSYDHLENEELKSIFFLCAQMGHQPLIMDLVKYCFGLGILEGVYSLGEARDRISTSIKKLKDSGLVLDGSSSIHFNMHDLVRDAALSIAQNEQNVFTLRNGKLNDWPELKRCTSISICNSDIIDELPNVMNCPQLKFFQIDNDDPSLKIPESFFKRMKKLRVLILTGFHLSSLPSSIKCLSDLRLLCLERCTLDHNLSIIGKLKKLRILSFSGSRIENLPAELKDLDKLQLLDISNCSIVTMIPPNLISRLTSLEELYVRKCFMEVSEEGERNQSQNSFISELKHLHQLQVVDLSIPCAEFFAKELFFDNLSDYKIEIGNFKTLSAGDFRMPNKYENFKSLALELKDDTDNIHSQTGIKLLFETVENLLLGELNGVQDVVNELNLNGFPHLKHFSIVNNPSIKYIINSKDLFYPQDVFPKLESLCLYKLKEIEMIYFSSGTEMICFSPFTDCSFTKLKTIKVEKCDQLKNLFSFCMVKLLASLETIGVSNCGSLEEIIKIPDNSDKIEFLKLMSLSLESLSSFTSFYTTVEGSSTNRDQIQITVMTPPLFGELVEIPNLENLNLISMNKIQKIWSDQPPSNFCFQNLIKLVVKDCQNLRYLCSLSVASSLRKLKGLFVSNCKMMEKIFSTEGNSADKVCVFPKLEEIHLDQMDELTDIWQAEVSADSFSSLTSVNIRSCHKLDKIFPSHMEGWFASLNSLKVSFCESVEVIFEIKDSQQVDASGGIDTNLQVVYVSELPKLEQVWSRDPGGILNFKKLQSIEMDDCERLRNVFPASVGKDVPKLEYMSVIECDGIVEIVACEDGSETNTEQLVFPELTDMELCYLSSIQHFYRGRHPIECPKLKKLSVGKCNEKLKTFGTGERSNEEDEAVMSAEKIFPNLEYLDIHFDEAQKWLLSNTVKHRMHRLKKLRLSEVNDGERLCQILYRMPNLEKLYLPMAKHLLKESSESRLGTLLQLKELDLWRSEIKDIGFEREPVLQRLELLSLFKCHKLRNLGPPSVSLAYLTNLKVEYCYGLRNLMASSTAKSLVQLKSMKIRKCCKLEEIVSDEGNEEEEQIVFGKLITIELEGLKKLKSFCSSKNYEFKFPSLEGLIVRKCPMMHTFTEGDARAPKLENTVTAKEEGKEEAKWQWEGDLNSTIQKGFNKLLESASTESSLSLIDSPLQVIWLDSRRIPKSCFSNLTELTVHGCQFLTDVVIPFYLLPFLTNLQQLQVSDCRSVKSIFDVKTAMGLGAAAFPRPLPFSLKKLTLEWLPKLENVWNEDPHGILTMQLLQHVKVKNCKCLTSVFPASLAKDLEKLVVKDCEGLIEIVAEDNADPREANLELTFPCPCVSSLKLQGLPKFKYFYYCSLQCDMFQTPTKDEMPTSNLQCLSLGEKGLEMIKRAEFQRNFLHKLQVLTLCFHIGSNVFPYEILQLAPNIEKLVVCDGSFKEIFCFDSLNVDEAGLLLQLKVLCLESLPELVSIGLENSWIQPLLGNLETLEVIGCSSLKDLVPSTVSFSNLTYLEVERCHCLLYLFTSSTARSLGQLKRMEIKWCGSIEEVVVSKEGDESHEEEIIFPQLNCLKLEYLSKLRSFYKGSLLSFPSLEELSVIRCEWMETLCPGTLKADKLVQVQLKESYSWRHSDPIKLENDLNSTMREAFWKKLWKSADTEFSIDLKDSPVQEIWLRLHSLHIPPHFCFPKLHTLIVDGCHFLSDAVLPFSLLPLLPNLKTLEVRNCDFVKIIFDVTTMGPLPFALKTLALCDLPNLENVWNSNVELTFPQVKSLALCDLPKLKYDILKPFTHLEPHTLNQVCIQKLTPNIEHLTLGQHELNMILSGEFQGNHLNELKVLALFFHIESDVFLQRVPNIEKLEVRDGSFKEIFCFDSLNVDEDGLVSQLKVICPDSLPELVSIGSENSGIVPFLRNLETLQVISCFSSINLVPCTVSFSNLTYLKVESCKSLLYLFTSSTARSLGQLKTMEISWCNSIEEIVSSTEEGDESDENEIIFQQLNCLKLEFLFKLRRFYKGSLSFPSLEEFTVWRCERMESLCAGTVKTDKLLQVNTNWGGDVIPLETDLNSAMQNR

Haro33 *Rps3b* coding sequence

>ATGGACGCTGTGTCATCCGCACTACTAGAGCCAGTAACTAATTCTGTGTTGGATCTGCTTAAAAAGCAACTGGATTACATCCGTTACAGGCGAAACTTTGATGAACTAGACGAGTGTGTTAAGCAGCTTAAACATAAAAAGGAGATAGTAGATCATCAATGTGAGGAAGCTGTCAAAAATGGACACGAAATTGAAGGTAAGGTTAGAGAATGGTTAGGGAAAGTGGGTAAATTTGAGACAGAAGTGGAGAAGTATTGGAACGATGATGGCCACAAAAAGACACGGTTTTCCAACTATTTATTTCCTTACTTTAGGCATAGACTAGGCAGACTAGCAAAGAAGATGGCAGTTGAGGGTAAAAAGATAACCGATGATTGCCCAAAGTCTGATGAAATTGCCTATAGGGTATACGTAACATCTAATTATGCCATTTTGTCTAATAATGACCTTATGGATTTTGGTTCTAGAAAATCCATAATGGAACAAATAATGGCAACACTTGTTGAAGATCCCACTGTGAAAATGATTGGAGTGTATGGACGAAGTGGGGTGGGTAAGAGCACTTTAATCAAAGCAATTGCTAAAATTGCTCGAGACAAGAAGTTGTTTAATGTGGTGGCTTTTTCAGAAATAACAGACAACCCCAATCTAAAACAAGTCCAGGAAGATATTGCTTACCCTTTGGGATTGAAATTGGAAGGAGAAGGTGAGAATGTAAGAGCTGATCATCTACGAAGGAGGTTAAAGAAAGAGAAAGAGAACACCCTTATAATCTTGGATGACCTTTGGGACAGATTAGACTTGAATAGGTTGGGAATTCCACTTGATGGTGATGTTGATGATAAACAGGGTCCCAAAGGGCCGACAAAAGAAAAATCTCTTGCTGATTATAAGGGTTGCAAAATTTTGCTAACTTCAAGGAAACGAAATGTATTAACGGATAAAATGGAAGTTAAATTAACTTTCTGTGTAGAGGAATTAGATGAAAAAGATGCTCTGAAGTTGTTTCGGAAGGAGGCTGCAATACAAGGTGAAATGTCCAAGTCTAAAAAAGAAATTGTTAAGAAGTATTGTGCTGGGTTACCTATGGCAATAGTTACAGTTGGAAGGGCATTAAGAGACAAGAGCGACTCAGAGTGGGAAAAACTTAAAAACCAAGAACTGGTGGGAGTTCAGAATCCGATGGAGATTTCTGTAAAAATGAGTTATGACCATCTAGAAAATGAGGAGCTCAAGTCCATTTTCTTTCTTTGTGCTCAAATGGGTCATCAACCCCTAATTATGGACTTGGTGAAGTATTGCTTTGGTTTGGGAATACTTGAAGGGGTCTACTCGCTTGGGGAAGCTCGGGACAGAATATCTACATCAATCAAAAAGCTGAAAGACTCAGGTTTGGTGTTGGATGGAAGTTCTAGTATTCATTTCAATATGCACGATCTGGTTCGAGATGCTGCTTTATCTATAGCACAGAACGAGCAAAATGTATTTACTTTGAGAAATGGGAAACTTAATGATTGGCCTGAACTCAAGAGGTGCACTTCTATTTCTATATGCAATAGTGATATCATTGATGAGCTTCCTAATGTTATGAACTGTCCTCAACTTAAATTTTTCCAAATTGACAATGATGATCCATCTTTAAAAATACCTGAGAGTTTTTTTAAGAGAATGAAAAAACTCAGAGTGTTAATATTGACTGGCTTTCATCTATCAAGCTTACCATCATCAATTAAGTGCCTATCAGACCTCAGATTGCTTTGTTTGGAGCGATGCACTTTAGATCACAACTTATCCATCATAGGGAAGCTGAAAAAATTAAGAATTCTCAGCTTTTCTGGATCTCGAATTGAAAATTTGCCAGCTGAGTTGAAGGACTTGGATAAACTACAATTACTAGACATCAGCAATTGTTCAATAGTCACCATGATTCCACCTAATCTTATATCAAGGTTGACTTCGTTGGAAGAGCTGTATGTAAGAAAGTGTTTCATGGAAGTGTCGGAGGAAGGAGAGAGAAACCAAAGTCAAAATTCATTTATTTCTGAACTAAAGCATTTGCATCAATTGCAAGTGGTGGACTTAAGCATTCCATGTGCTGAATTTTTTGCAAAGGAATTGTTCTTTGACAACTTAAGTGATTACAAGATTGAGATTGGGAACTTCAAAACTCTTTCAGCTGGAGATTTCAGAATGCCTAATAAGTATGAAAATTTCAAATCTTTGGCATTGGAGCTGAAGGATGACACTGACAATATTCACTCTCAGACAGGAATAAAGTTGTTGTTTGAAACAGTTGAAAATTTGTTGTTGGGAGAGCTGAATGGTGTTCAAGATGTTATTAATGAGTTGAATTTGAATGGATTTCCACATCTGAAACACTTTTCCATCGTAAACAACCCTAGCATCAAATATATCATCAACTCAAAGGATTTGTTTTATCCTCAGGATGTTTTTCCCAAGTTGGAATCTCTATGCCTCTACAAACTAAAAGAGATAGAGATGATATACTTTAGTTCAGGTACAGAGATGATATGCTTTAGTCCATTTACAGATTGCTCATTCACCAAATTAAAAACCATCAAGGTCGAGAAGTGTGATCAATTGAAGAATCTTTTCTCCTTTTGCATGGTTAAATTGCTTGCGAGTCTTGAAACAATTGGTGTTTCCAATTGTGGTTCTTTAGAGGAGATCATTAAAATACCAGACAATTCTGATAAGATTGAGTTTCTTAAGTTGATGTCTTTGTCACTTGAATCATTATCATCATTCACTAGTTTTTATACCACAGTAGAGGGGTCTTCTACAAACAGAGATCAGATACAAATTACTGTTATGACTCCTCCTCTTTTTGGTGAACTGGTTGAAATACCAAACTTAGAGAACTTGAATTTAATCTCAATGAACAAGATCCAGAAGATATGGAGCGACCAGCCCCCGTCAAACTTCTGCTTTCAAAACTTAATAAAATTAGTTGTGAAAGATTGTCAAAATTTGAGATATTTGTGTTCATTGTCCGTGGCCAGCAGTTTGAGGAAACTGAAAGGCCTCTTTGTAAGCAACTGTAAAATGATGGAGAAGATTTTTAGCACAGAAGGAAATAGTGCAGACAAGGTTTGCGTCTTTCCTAAGTTGGAGGAAATTCACCTCGACCAAATGGATGAGTTAACAGACATATGGCAAGCTGAAGTGAGTGCTGATTCCTTTTCTAGTCTCACTTCTGTGAACATTCGTAGTTGTCATAAACTAGACAAAATTTTTCCGAGTCACATGGAAGGATGGTTTGCGAGTTTGAACAGCTTGAAGGTTTCTTTTTGTGAGTCAGTGGAAGTGATTTTTGAAATCAAAGATTCTCAGCAAGTAGATGCATCTGGTGGGATAGACACAAATTTGCAGGTTGTTTATGTAAGTGAACTCCCAAAGTTGGAGCAGGTGTGGAGCAGGGATCCAGGAGGAATTCTTAACTTCAAAAAACTGCAGAGTATAGAGATGGATGATTGTGAAAGACTGAGGAATGTATTTCCAGCTTCTGTGGGCAAAGATGTTCCAAAGCTTGAATACATGTCGGTCATAGAGTGTGATGGAATTGTGGAAATTGTTGCCTGTGAAGATGGATCCGAAACAAACACTGAACAATTAGTGTTTCCTGAACTAACCGACATGAAATTATGTTACCTATCAAGCATCCAGCATTTCTACAGGGGGAGACATCCTATAGAGTGTCCAAAATTGAAGAAGTTGTCAGTAGGGGGATGTAACGAGAAGCTAAAAACATTCGGAACCGGAGAAAGGAGCAATGAAGAAGATGAAGCAGTTATGTCAGCTGAAAAGATATTCCCCAACTTGGAGTTTTTGAATACTCGCTTTGACGAAGCACAAAAGTGGTTATTGAGCAACACTGTGAAGCATCGAATGCACCGTTTAAAAGAGCTTCACTTGCACGAAGTTAATGATGGTGAACGTCTCTGTCAAATTCTGTACAGAATGCCAAATCTAGAAAAGTTATACTTGCCGATGGCTAAACATTTGCTTAAAGAGTCGTCGGAGTCCCGTTTGGGAACCCTATTACAGCTGAAGGAATTGGATTTGTGGAGGTCGGAGATAAAGGATATAGGATTTGAACGAGAACCAGTTCTACAGAGACTAGAGCTTTTGAGCTTATTTAAGTGCCATAAATTGAGGAATTTGGGTCCTCCCTCGGTATCATTGGCTTACTTGACAAATTTGAAAGTAGAGTATTGTTATGGATTAAGGAATTTAATGGCATCCTCAACGGCAAAAAGCTTGGTTCAACTTAAGTCCATGAAGATAAGAAAATGTTGTAAATTAGAGGAAATAGTAAGCGATGAGGGAAATGAAGAAGAAGAGCAAATAGTGTTTGGCAAATTGATTACTATAGAACTTGAGGGGCTAAAAAAGCTGAAAAGTTTTTGCAGTTCCAAGAACTATGAATTCAAATTCCCGTCATTGGAAGGATTGATTGTGAGAAAATGCCCAATGATGCATACATTCACGGAGGGTGACGCAAGAGCACCAAAGTTAGAAAACACAGTTACTGCTAAAGAAGAAGGAAAAGAGGAAGCCAAATGGCAGTGGGAAGGAGACTTGAATTCCACCATACAAAAAGGTTTCAACAAGCTTTTGGAGTCTGCAAGTACTGAATCATCTCTTAGTCTCATAGATAGCCCACTACAAGTGATATGGCTTGACTCACGGCGGATCCCAAAGTCGTGCTTCAGTAACTTGACGGAATTGACTGTGCACGGATGCCAATTTTTAACAGATGTTGTCATACCCTTCTATTTACTTCCTTTCTTAACTAATTTGCAACAATTACAAGTCTCGGACTGTCGTTCTGTGAAAAGCATATTTGACGTGAAAACAGCTATGGGATTGGGAGCAGCAGCCTTCCCTAGACCTCTCCCTTTTTCCCTCAAGAAATTGACTTTAGAGTGGCTGCCAAAACTGGAGAATGTCTGGAATGAAGATCCTCATGGAATTCTAACCATGCAACTTCTACAACATGTAAAGGTTAAAAATTGTAAATGCCTTACAAGTGTGTTTCCGGCATCATTAGCCAAAGATCTTGAAAAACTAGTTGTCAAAGACTGTGAGGGATTGATAGAAATTGTTGCAGAGGATAATGCAGATCCAAGAGAAGCAAATCTGGAGCTTACGTTCCCTTGTCCCTGTGTGAGCTCATTGAAACTACAAGGTTTGCCCAAGTTCAAGTATTTTTACTACTGCTCACTGCAGTGTGACATGTTCCAGACACCTACCAAGGATGAAATGCCTACATCCAACTTACAGTGCCTGTCACTCGGTGAAAAAGGACTGGAGATGATCAAGCGTGCAGAATTTCAGAGAAACTTCTTACACAAGTTACAAGTTCTTACTCTGTGCTTTCATATTGGGTCGAATGTATTTCCATATGAAATTCTACAACTGGCGCCCAATATAGAGAAGCTTGTGGTGTGTGATGGTTCCTTCAAGGAGATTTTCTGCTTTGATAGTCTTAATGTGGATGAGGCTGGACTCCTATTACAGCTCAAAGTCTTATGCTTGGAGTCCCTTCCAGAGCTTGTTTCCATTGGGTTAGAGAACTCTTGGATTCAGCCCTTACTGGGAAATCTAGAAACCTTGGAAGTAATAGGTTGTTCTAGTTTAAAAGACTTGGTACCATCTACAGTGTCTTTTTCCAATCTGACATATTTGGAAGTAGAAAGATGCCATTGCCTGCTATATTTGTTCACATCCTCCACAGCAAGAAGTTTGGGTCAACTCAAAAGAATGGAGATAAAATGGTGTGGTTCAATTGAAGAGGTAGTAGTCTCTAAGGAGGGGGATGAATCACATGAGGAGGAGATTATATTTCCGCAGCTCAATTGTTTGAAACTTGAATATTTATCAAAGCTGAGAAGCTTCTATAAAGGAAGTTTATTAAGTTTCCCATCATTGGAGGAATTGTCAGTAATCCGTTGCGAGTGGATGGAAACATTATGTCCAGGTACCCTTAAAGCAGACAAGTTGGTTCAAGTTCAACTTAAGGAGAGTTATTCTTGGAGGCACTCAGATCCTATCAAATTGGAAAATGACCTGAACTCTACCATGCGGGAGGCATTTTGGAAAAAGTTATGGAAGTCTGCAGATACGGAATTCAGTATTGACCTCAAAGATAGCCCAGTACAAGAGATATGGCTTAGGCTTCACTCACTGCATATCCCCCCACACTTCTGCTTCCCTAAGTTAGACACCTTGATTGTGGACGGCTGCCATTTTTTATCAGATGCGGTCTTACCCTTCTCTTTACTTCCTTTATTACCTAATTTGAAAACATTGGAAGTTCGAAACTGTGATTTTGTGAAAATCATATTTGATGTGACAACTATGGGACCACTCCCTTTTGCCCTCAAGACATTGGCACTGTGTGATCTGCCAAATCTGGAGAATGTTTGGAATTCAAATGTTGAGCTTACGTTCCCCCAAGTCAAGTCATTGGCACTCTGTGATCTGCCAAAGTTAAAGTATGACATCTTGAAGCCATTTACACATCTAGAACCACATACTCTAAATCAAGTCTGTATTCAAAAGCTTACACCCAACATAGAGCACCTGACACTCGGTGAACATGAACTCAACATGATTTTGAGTGGAGAATTCCAGGGAAACCACTTAAACGAGTTAAAAGTGCTTGCTCTGTTCTTTCATATTGAATCCGATGTATTTCTACAACGGGTGCCCAATATAGAGAAGCTTGAGGTGCGTGATGGTTCCTTCAAAGAGATTTTCTGCTTTGATAGCCTTAATGTGGATGAGGATGGATTGGTTTCACAGCTGAAAGTGATATGCCCGGACTCCCTTCCAGAGCTTGTTTCCATTGGGTCAGAGAACTCTGGGATTGTGCCCTTTCTCAGAAATCTAGAAACATTGCAAGTAATCAGCTGTTTCAGTTCAATAAATCTGGTACCATGCACAGTGTCTTTTTCCAATCTGACATATTTGAAAGTAGAAAGTTGCAAGAGTCTGCTATATTTGTTCACATCCTCAACAGCAAGAAGTTTGGGTCAACTCAAAACAATGGAGATAAGTTGGTGTAATTCAATTGAAGAGATAGTGTCTTCAACAGAGGAAGGGGATGAATCAGATGAGAATGAGATAATATTTCAGCAGCTCAATTGTTTGAAACTTGAATTTTTATTTAAGCTGAGAAGGTTCTACAAAGGGAGTTTAAGTTTCCCGTCCTTGGAGGAATTCACAGTATGGCGTTGCGAGAGGATGGAAAGTTTGTGTGCAGGTACAGTCAAAACAGACAAGCTGTTACAAGTGAAAACTAATTGGGGCGGAGATGTTATCCCATTGGAAACTGATCTGAACTCTGCCATGCAAAACCGATAG

Haro33 *Rps3b* protein sequence

>MDAVSSALLEPVTNSVLDLLKKQLDYIRYRRNFDELDECVKQLKHKKEIVDHQCEEAVKNGHEIEGKVREWLGKVGKFETEVEKYWNDDGHKKTRFSNYLFPYFRHRLGRLAKKMAVEGKKITDDCPKSDEIAYRVYVTSNYAILSNNDLMDFGSRKSIMEQIMATLVEDPTVKMIGVYGRSGVGKSTLIKAIAKIARDKKLFNVVAFSEITDNPNLKQVQEDIAYPLGLKLEGEGENVRADHLRRRLKKEKENTLIILDDLWDRLDLNRLGIPLDGDVDDKQGPKGPTKEKSLADYKGCKILLTSRKRNVLTDKMEVKLTFCVEELDEKDALKLFRKEAAIQGEMSKSKKEIVKKYCAGLPMAIVTVGRALRDKSDSEWEKLKNQELVGVQNPMEISVKMSYDHLENEELKSIFFLCAQMGHQPLIMDLVKYCFGLGILEGVYSLGEARDRISTSIKKLKDSGLVLDGSSSIHFNMHDLVRDAALSIAQNEQNVFTLRNGKLNDWPELKRCTSISICNSDIIDELPNVMNCPQLKFFQIDNDDPSLKIPESFFKRMKKLRVLILTGFHLSSLPSSIKCLSDLRLLCLERCTLDHNLSIIGKLKKLRILSFSGSRIENLPAELKDLDKLQLLDISNCSIVTMIPPNLISRLTSLEELYVRKCFMEVSEEGERNQSQNSFISELKHLHQLQVVDLSIPCAEFFAKELFFDNLSDYKIEIGNFKTLSAGDFRMPNKYENFKSLALELKDDTDNIHSQTGIKLLFETVENLLLGELNGVQDVINELNLNGFPHLKHFSIVNNPSIKYIINSKDLFYPQDVFPKLESLCLYKLKEIEMIYFSSGTEMICFSPFTDCSFTKLKTIKVEKCDQLKNLFSFCMVKLLASLETIGVSNCGSLEEIIKIPDNSDKIEFLKLMSLSLESLSSFTSFYTTVEGSSTNRDQIQITVMTPPLFGELVEIPNLENLNLISMNKIQKIWSDQPPSNFCFQNLIKLVVKDCQNLRYLCSLSVASSLRKLKGLFVSNCKMMEKIFSTEGNSADKVCVFPKLEEIHLDQMDELTDIWQAEVSADSFSSLTSVNIRSCHKLDKIFPSHMEGWFASLNSLKVSFCESVEVIFEIKDSQQVDASGGIDTNLQVVYVSELPKLEQVWSRDPGGILNFKKLQSIEMDDCERLRNVFPASVGKDVPKLEYMSVIECDGIVEIVACEDGSETNTEQLVFPELTDMKLCYLSSIQHFYRGRHPIECPKLKKLSVGGCNEKLKTFGTGERSNEEDEAVMSAEKIFPNLEFLNTRFDEAQKWLLSNTVKHRMHRLKELHLHEVNDGERLCQILYRMPNLEKLYLPMAKHLLKESSESRLGTLLQLKELDLWRSEIKDIGFEREPVLQRLELLSLFKCHKLRNLGPPSVSLAYLTNLKVEYCYGLRNLMASSTAKSLVQLKSMKIRKCCKLEEIVSDEGNEEEEQIVFGKLITIELEGLKKLKSFCSSKNYEFKFPSLEGLIVRKCPMMHTFTEGDARAPKLENTVTAKEEGKEEAKWQWEGDLNSTIQKGFNKLLESASTESSLSLIDSPLQVIWLDSRRIPKSCFSNLTELTVHGCQFLTDVVIPFYLLPFLTNLQQLQVSDCRSVKSIFDVKTAMGLGAAAFPRPLPFSLKKLTLEWLPKLENVWNEDPHGILTMQLLQHVKVKNCKCLTSVFPASLAKDLEKLVVKDCEGLIEIVAEDNADPREANLELTFPCPCVSSLKLQGLPKFKYFYYCSLQCDMFQTPTKDEMPTSNLQCLSLGEKGLEMIKRAEFQRNFLHKLQVLTLCFHIGSNVFPYEILQLAPNIEKLVVCDGSFKEIFCFDSLNVDEAGLLLQLKVLCLESLPELVSIGLENSWIQPLLGNLETLEVIGCSSLKDLVPSTVSFSNLTYLEVERCHCLLYLFTSSTARSLGQLKRMEIKWCGSIEEVVVSKEGDESHEEEIIFPQLNCLKLEYLSKLRSFYKGSLLSFPSLEELSVIRCEWMETLCPGTLKADKLVQVQLKESYSWRHSDPIKLENDLNSTMREAFWKKLWKSADTEFSIDLKDSPVQEIWLRLHSLHIPPHFCFPKLDTLIVDGCHFLSDAVLPFSLLPLLPNLKTLEVRNCDFVKIIFDVTTMGPLPFALKTLALCDLPNLENVWNSNVELTFPQVKSLALCDLPKLKYDILKPFTHLEPHTLNQVCIQKLTPNIEHLTLGQHELNMILSGEFQGNHLNELKVLALFFHIESDVFLQRVPNIEKLEVRDGSFKEIFCFDSLNVDEDGLVSQLKVICPDSLPELVSIGSENSGIVPFLRNLETLQVISCFSSINLVPCTVSFSNLTYLKVESCKSLLYLFTSSTARSLGQLKTMEISWCNSIEEIVSSTEEGDESDENEIIFQQLNCLKLEFLFKLRRFYKGSLSFPSLEEFTVWRCERMESLCAGTVKTDKLLQVKTNWGGDVIPLETDLNSAMQNR
